# Microglial activation without peripheral immune cell infiltration characterises mouse and human cerebral small vessel disease

**DOI:** 10.1111/nan.13015

**Published:** 2024-11-14

**Authors:** Tushar Deshpande, Melanie‐Jane Hannocks, Kishan Kapupara, Sai Kiran Reddy Samawar, Lydia Wachsmuth, Cornelius Faber, Colin Smith, Joanna Wardlaw, Lydia Sorokin

**Affiliations:** ^1^ Institute of Physiological Chemistry and Pathobiochemistry and Cells‐in‐Motion Interfaculty Centre (CiMIC) University of Muenster Muenster Germany; ^2^ Clinic of Radiology University of Muenster Muenster Germany; ^3^ Centre for Clinical Brain Sciences University of Edinburgh Edinburgh UK; ^4^ Centre for Clinical Brain Sciences, UK Dementia Research Institute University of Edinburgh Edinburgh UK

**Keywords:** cerebral small vessel disease, microglial activation, mouse models of SVD, peripheral immune cells, postmortem MRI‐histopathology correlations

## Abstract

**Aims:**

Cerebral small vessel diseases (SVDs) involve diverse pathologies of the brain's small blood vessels, leading to cognitive deficits. Cerebral magnetic resonance imaging (MRI) reveals white matter hyperintensities (WMHs), lacunes, microbleeds and enlarged perivascular spaces in SVD patients. Although correlations of MRI and histopathology help to understand the pathogenesis of SVD, they do not explain disease progression. Mouse models, both genetic and sporadic, are valuable for studying SVD, but their resemblance to clinical SVD is unclear. The study examined similarities and differences between mouse models of SVDs and human nonamyloid SVD specimens.

**Methods:**

We analysed four mouse models of SVD (hypertensive BPH mice, Col4a1 mutants, Notch3 mutants and Htra1^−/−^ mice) at different stages for changes in myelin, blood‐brain barrier (BBB) markers, immune cell populations and immune activation. The observations from mouse models were compared with human SVD specimens from different regions, including the periventricular, frontal, central and occipital white matter. Postmortem MRI followed by MBP immunostaining was used to identify white matter lesions (WMLs).

**Results:**

Only Notch3 mutant and hypertensive BPH mice showed significant changes in myelin basic protein (MBP) immunostaining, correlating with MRI patterns. These changes were linked to altered microglial morphology and focal plasma protein staining around blood vessels, without peripheral immune cell infiltration. In human specimens, both normal‐appearing white matter (NAWM) and WMLs lacked peripheral cell infiltration. However, WMLs displayed altered microglial morphology, reduced myelin staining and occasional fibrinogen staining around arterioles and venules.

**Conclusions:**

Our data show that *Notch3* mutants and hypertensive BPH/2J mice recapitulate several features of human SVD, including microglial activation, focal sites of demyelination and perivascular plasma protein leakage without peripheral immune cell infiltration.

## Graphical abstract

Human and mouse cerebral small vessel diseases present microglial activation without peripheral immune cell infiltration. The areas of microglial activation coincide with myelin lesions rather than focal extravascular serum proteins. Notch3 mutants and hypertensive BPH/2J mice recapitulate several features of human SVD.

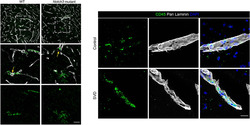

**Key Points**

- A comparison of mouse and human cerebral small vessel disease (SVD) brains identifies white matter changes, microglial activation and focal perivascular serum protein accumulation as the common pathological features.
- Notch3 mutants and hypertensive BPH/2J mice show early white matter changes similar to those in human SVD.
- Extravascular plasma proteins, rather than peripheral immune cells, are present in diseased brains.
- Postmortem MRI‐histopathology correlations reveal colocalization of activated microglia and white matter lesions.

## INTRODUCTION

Cerebral small vessel diseases (SVDs) are pathological changes in cerebral small vessels that correlate with cognitive decline. Magnetic resonance imaging (MRI) features of SVD include white matter hyperintensity (WMH), lacunar infarcts, microbleeds and enlarged perivascular spaces [1, 2, 3]. The sporadic forms of SVD include arteriolosclerosis, lipohyalinosis (associated with ageing, hypertension and other vascular risk factors) and cerebral amyloid angiopathy. There are monogenic forms of SVD that include mutations in the human *NOTCH3* gene causing cerebral dominant arteriopathy with cerebral infarcts and leukoencephalopathy (CADASIL) [4], mutations in the human *COL4A1 OR COL4A2* gene causing haemorrhagic SVD [5, 6] and mutations in the gene encoding the serine protease *HTRA1*, causing cerebral recessive arteriopathy with subcortical infarctions and leukoencephalopathy (CARASIL) [7].

WMHs are among the most common features of SVDs [8], and the aetiology of these lesions is not fully understood. Imaging studies have indicated fluid accumulation, especially in the periventricular white matter [9] and other white matter regions, which can result in MRI hyperintensity in deep subcortical structures, especially at the early stages of the disease. Autopsy studies revealed variable degrees of demyelination and axonal loss associated with white matter lesions (WMLs) [10]. As WMHs can be small, postmortem identification of these lesions is challenging. Thus, postmortem MRI of histology blocks with subsequent histopathology correlations help define the nature of WMHs [10, 11]. However, the study of the dynamic progression of WMHs at the histological level requires animal models of SVD. Although mouse and rat models of hypertension have been developed, the extent to which they resemble human sporadic SVD is not clear [12]. Monogenic forms of SVD can be studied using mouse models involving specific mutations of the causative genes. *Notch3* mutant mice have been characterised and shown to be similar to human CADASIL [13], while mutations in *Col4a1* genes result in intracerebral haemorrhages in mice and humans [5], and patients with *Col4a1* mutations display white matter defects [14]. *Htra1* knockout mice (*Htra1* ^*−/−*^) have been postulated to recapitulate features of human CARASIL [15]. Hypertension is a leading risk factor for dementia, and recent studies have revealed the BPH hypertensive mouse model shares several characteristics with human SVD [16]. All these mouse models present variable degrees of white matter changes. Comparing their pathology with each other and with human SVD will help assess their potential in studies of disease progression.

White matter changes in SVD have been postulated to be of ischaemic origin [17, 18]. Such changes are also observed in multiple sclerosis (MS) patients and can result in the misdiagnosis of SVD as MS or vice versa [19, 20]. Although the neuroinflammatory mechanisms of MS are well known, whether they are also relevant to SVD is poorly understood, despite perivascular inflammation being described in the 1800s [21]. Systemic inflammatory arthropathies are associated with increased SVD lesions compared to age‐matched healthy controls and patients with stroke [22, 23]. However, the nature of immune cells involved in inflammation and their contribution to this disease are unknown. The use of anti‐inflammatory drugs has been proposed for the clinical management of SVDs [24], but clinical trials involving such approaches have had limited success [25]. In this context, it is important to determine the exact role peripheral immune cells play in SVD pathogenesis, especially in white matter lesions, where the infiltration of peripheral immune cells and extravasation of serum proteins have not been systematically investigated. Therefore, colocalisation of immune cells and serum proteins with white matter lesions warrants further investigation into their causal link. In this study, we systematically evaluated four mouse models of SVD (hypertensive BPH mice, *Col4a1* mutants, *Notch3* mutants and mice lacking the HtrA serine protease *Htra1* ^−/−^) for the infiltration of peripheral immune cells after postmortem MRI of the brain and for the activation of resident immune cell populations and blood–brain barrier (BBB) integrity. We compared our findings to brain autopsy specimens from patients with nonamyloid SVD using postmortem MRI‐guided histopathology. The periventricular white matter and other white matter regions were examined for signs of inflammation and myelin damage. Our data suggest that SVD‐induced white matter damage involves the activation of resident immune cells with little or no infiltration from peripheral immune cells unless there is a haemorrhagic lesion. Mouse models of SVD recapitulate many features of human SVD, with the exception of the *Htra1* ^−/−^ mouse model.

## METHODS

### Mouse models

Spontaneously hypertensive BPH (BPH/2J, Jackson Lab 003005, referred to as hypertensive mice) mice [26, 27], *Col4a1* transgenic (*Col4a1* ^*+/G498V*^, referred to as the *Col4a1* mutant) mice [28], *Notch3* transgenic (*Notch3* ^R169C/R169C,^ referred to as the *Notch3* mutants, also known as CADASIL mice) mice [13] and *Htra1* knockout (*Htra1* ^−/−^, also known as CARASIL) [15] mice were maintained according to local Animal Welfare guidelines. Brains were collected at different time points (6 and 8 months for BPH mice; 6, 12 and 20 months for *Notch3* mutants; 6 months for *Col4a1* mutant mice; and 6 and 12 months for *Htra1* ^*−/−*^ mice). Age‐matched normotensive BPN mice were used as controls for the BPH mice. For other mouse models, age‐matched littermates were used as controls. Each group comprised equal numbers of male and female mice (Table 1).

**TABLE 1:** Mouse SVD models employed and time points analysed.

Mouse model	Time points
BPH	6 and 8 months
*Notch3* mutants	6, 12 and 20 months
*Col4a1* ^*+/−*^ mutant	6 months
*Htra1* ^−/−^	6 and 12 months

Abbreviation: SVD, small vessel disease.

### Mouse brain MRI

Mouse brains were fixed overnight at 4°C in 4% paraformaldehyde, followed by washing and immersion in 2mM Magnevist (Bayer Vital GmbH, Leverkusen, Germany) for 24 hours. Whole brains or two halves of the brain (from transgenic/hypertensive and corresponding wild‐type mice, cut in the sagittal plane at the midline) were embedded in 1% agar containing 2mM Magnevist. MRI was performed at 9.4 T using a 0.7‐T/m gradient insert and a helium‐cooled mouse brain surface coil operated with ParaVision 5.1 software (Bio‐Spec 94/20; CryoProbe, Bruker BioSpin MRI GmbH, Ettlingen, Germany). Sagittal or coronal 3D T2‐weighted anatomical images were obtained with a rapid acquisition with relaxation enhancement (RARE) protocol: TR 2000 ms, TE effective 73 ms, RARE factor 24, two averages, field of view (FOV) 16 × 16 × 12 mm^3^, matrix 384 × 384 × 256 and voxel size 40 × 40 × 500 μm^3^. Diffusion tensor imaging (DTI) in the coronal plane (FOV 16 × 14, matrix 160 × 140, in‐plane resolution 100 × 100 μm^2^, 36 slDices, slice thickness 200 μm) was performed with an eight‐segment echo planar imaging (EPI) protocol (TR/TE 9000/34 ms) with 30 diffusion directions (*b* = 1000 s/mm^2^, diffusion gradient duration 4 ms, diffusion gradient separation 10 ms, five B0 images) and eight averages. Fractional anisotropy (FA) maps were calculated with the monoexponential fitting function implemented in PV5.1. Images were analysed with ImageJ (version 1.46r) for the mean FA and % area occupied above the FA threshold (0.2). Serial slices of the corpus callosum and striatum in the dorsal‐ventral direction were used for the analyses.

### Mouse brain immunofluorescence staining

Vibratome sections (60 μm) of the fixed brains or cryosections (10 μm) of the unfixed brains were used for immunostaining. Cryosections were fixed in methanol (10 min) before staining. The sections were blocked for 2 h at room temperature in 1% BSA with 0.5% Triton‐X100 and subsequently incubated overnight at 4°C with the primary antibodies listed in Table 2. After washing in PBS, the sections were incubated with secondary antibody solutions (donkey anti‐rat‐Alexa Fluor 488, donkey anti‐rabbit‐Cy3, donkey anti‐hamster‐Cy5 or donkey anti‐rat‐Cy5) (Molecular Probes) for 1 h at room temperature (RT). Nuclei were stained with DAPI. Sections were mounted in Elvanol [29] and imaged using a Zeiss confocal laser scanning microscope (LSM 700).

**TABLE 2:** List of primary antibodies.

Target	Antibody species (clone or ab name)	Source or reference	Concentration
Myelin basic protein	chicken anti‐bovine	Thermo Fisher PA1‐10008	1:500
CD45	rat anti‐mouse (30G12)	Ralph, 1984 #1593	NA
Pan‐Laminin	rabbit anti‐mouse (455)	{Sorokin, 1997 #930}	1:2000
Iba1	Rabbit anti‐mouse	Wako 019‐19741	1 μg/mL
P2RY12 (purinergic receptor)	Rabbit anti‐human	Sigma HPA013796	1 μg/mL
TMEM119	Rabbit anti‐mouse	Abcam ab209064	0.3 μg/mL
IgG	Cy3 goat anti‐mouse	Dianova 115‐165‐062	1.5 μg/mL
Aquaporin‐4	Rabbit anti‐rat	Millipore AB2218	1 μg/mL
PECAM‐1 (CD31)	Hamster anti‐mouse (2H8)	Abcam ab119341	2 μg/mL
Fibrinogen	Sheep anti‐human fibrinogen	Bio‐Rad 4440‐8004	5 μg/mL
α‐Smooth muscle actin	Cy3 mouse anti‐mouse (1A4)	Sigma C6198	3.6 μg/mL

### Human brain specimens

Formalin‐fixed paraffin‐embedded histology blocks from 30 nonamyloid SVD and control cohort brain specimens were obtained from the Edinburgh Brain Bank. To avoid the inclusion of Alzheimer's and Wallerian degeneration, the following criteria were employed in sample selection: minimal Alzheimer's disease neuropathologic change and absence of lacunar and cortical infarcts. For 20 of the nonamyloid SVD samples, fresh frozen tissue blocks were obtained from the contralateral hemisphere of the same brain. In total, seven periventricular white matter (PWM), six frontal white matter (FWM), six central white matter (CWM), five occipital white matter, four Brodmann area 44/45 (BA44/45) and two thalamus region samples were examined from 30 individuals (Supplementary data, Table 3).

### Human brain MRI

Postmortem scans of brain blocks were performed as described previously [11]. Briefly, 2 cm × 2 cm × 1 cm brain coronal sections were fixed in 10% formalin for 24–72 h before overnight scanning in a 7‐T small‐bore rodent MRI scanner (Agilent Technologies, Yarnton, UK) supplied with a 400‐mT/m gradient insert. The resultant T2‐weighted images had a field of view of 60 × 60 mm^2^ and a resolution of 0.23 × 0.23 × 1.0 mm^3^.

### Human brain immunofluorescence staining

Formalin‐fixed paraffin brain sections (10 μm) were deparaffinised in xylene and rehydrated in serial concentrations of ethanol (100%, 90%, 70%, 50%). The sections were subjected to antigen retrieval in citrate buffer, pH 6, in a pressure steamer for 30 min. The sections were then washed and blocked for 1 h at RT in 1% BSA plus 0.1% Triton‐X100. Frozen brain sections were fixed in −20°C methanol for 10 min, followed by blocking and staining, as for mouse samples.

### Image analysis

Human MR images were analysed with Fiji (version 2.3.051), while mouse MR images were analysed using ImageJ (version 1.46r). Postmortem mouse MR images were analysed according to the scheme shown in Figure S1. Regions of interest (ROIs) were selected for analysis from serial sections of the anterior mouse brain showing the striatum and corpus callosum. For mouse MRI, mean FA values were measured in the ROI, and the 95% confidence interval was determined using the standard deviation of the FA values of all the pixels in that region. To calculate the area using FA maps, the images were thresholded at 0.2.

Myelin basic protein (MBP) staining and T2 MRI were employed to assess white matter integrity in human samples. To quantify the WMH burden in human SVD samples, T2‐weighted images were thresholded using the Otsu algorithm [30]. In some cases, thresholds were manually adjusted (± 5%) to match the known grey and white matter areas. Control samples were defined as having less than 10% WMH. The calculated WMH was matched with neuropathological assessment by the brain bank, which included assessment of white matter pallor on haematoxylin and eosin and Luxol fast blue stained sections. For the analysis of immunofluorescent images of both human and mouse brains, the Zen blue program (Zeiss, Version 3.4) was used. Automatic thresholding using either the Otsu or Triangle algorithm [31] was employed in all image analyses. For Iba1 staining, signal intensity above the threshold set by the Otsu algorithm was considered for the analysis. To visualise and quantify MBP inside microglia, Imaris (version 9.9) was used.

### Statistical analysis

Statistical analyses were performed using the GraphPad Prism. All values are expressed as the means ± SDs. FA values are expressed as the mean ± 95% confidence interval. Human data are shown as medians with interquartile ranges. Normality was tested using the Shapiro–Wilk test. Nonnormally distributed groups were compared using the Mann–Whitney test. Two‐sided Student's *t*‐tests were applied to normally distributed data. The numbers of primary microglial branches were analysed using the Mann–Whitney test, and microglial circularity was analysed using Welch's t‐test. Correlations were analysed using the Pearson correlation coefficient. *p* < 0.05 was considered to indicate statistical significance.

## RESULTS

### Hypertensive BPH and *Notch3* mutant mice exhibit early white matter changes

**FIGURE 1:**
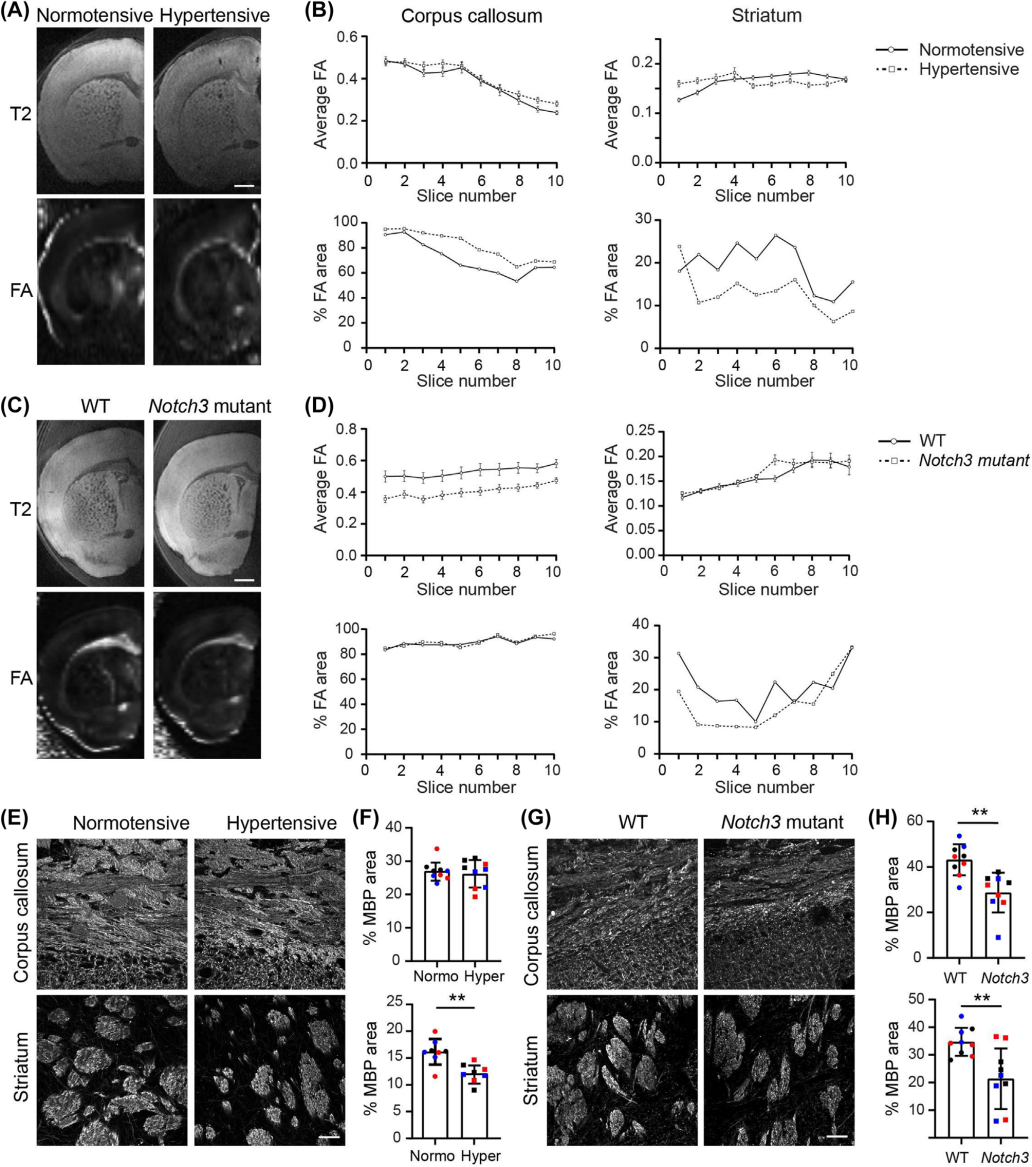
White matter integrity in mouse models of SVD. (A, C) MRI of fixed brains and (B, D) associated quantification of fractional anisotropy (FA) maps for (A, B) hypertensive and normotensive BPH/2J and (C, D) WT and *Notch3* mutant (CADASIL) brains. The upper panels in A and C are T2‐weighted images; the lower panels are FA maps; the scale bars are 1 mm. In B and D, the *X* axes represent the number of slices from dorsal to ventral (B) or from lateral to sagittal sulcus (D) with a slice thickness of 200 μm. The upper graphs show the average FA values in the corpus callosum and striatum; the lower graphs show the % area above the threshold in the FA maps. (E, G) Myelin basic protein (MBP) immunofluorescence staining of the corpus callosum and striatum of (E) normotensive and hypertensive and (G) WT and *Notch3* mutant brains; scale bars are 50 μm, and (F, H) corresponding quantification; data shown are means ± SDs from 3 to 6 sections/mouse for three mice/genotype; individual mice are colour‐coded. The data were analysed by the Mann–Whitney *U* test *p < 0.05, ***p < 0.001. MRI, magnetic resonance imaging; SVD, small vessel disease; WT, wild‐type.

High‐resolution T2‐weighted MRI scans were employed to detect broad structural changes, revealing no overt differences from control mice in the four mouse SVD models at any of the stages investigated (Figure 1A,C; Table 1). FA maps were quantified for mean intensity, and the percent area above the threshold (0.2) was used as a marker of white matter microstructure integrity. Lower FA values, indicating myelin defects, were detected only in hypertensive BPH/2J and *Notch3* mutant (CADASIL) mice at 6 months (Figure 1B,D). The % area above the threshold, denoting normally myelinated areas, was lower in these mice, particularly in the striatum. Parallel immunostaining for MBP confirmed a reduced myelinated area mainly in the striatum of hypertensive BPH mice and in the striatum and corpus callosum of *Notch3* mutants (Figure 1E–H). The corresponding MRI and immunofluorescence staining data for *Col4a1* mutants, which showed no defects in myelinated areas, are shown in Figure S2A–D. Six‐month‐old *Col4a1* mutant mice showed hypointense signals in T2 maps in the interbrain region (Figure S3A), indicating microhaemorrhages. This observation was confirmed by IgG immunostaining (Figure S3B,C), consistent with earlier reports [28]. *Htra1* ^−/−^ (CARASIL) mice did not show any significant changes in MBP staining (Figure S4A,B).

### No peripheral immune cell infiltration was detected in any of the mouse SVD models

**FIGURE 2:**
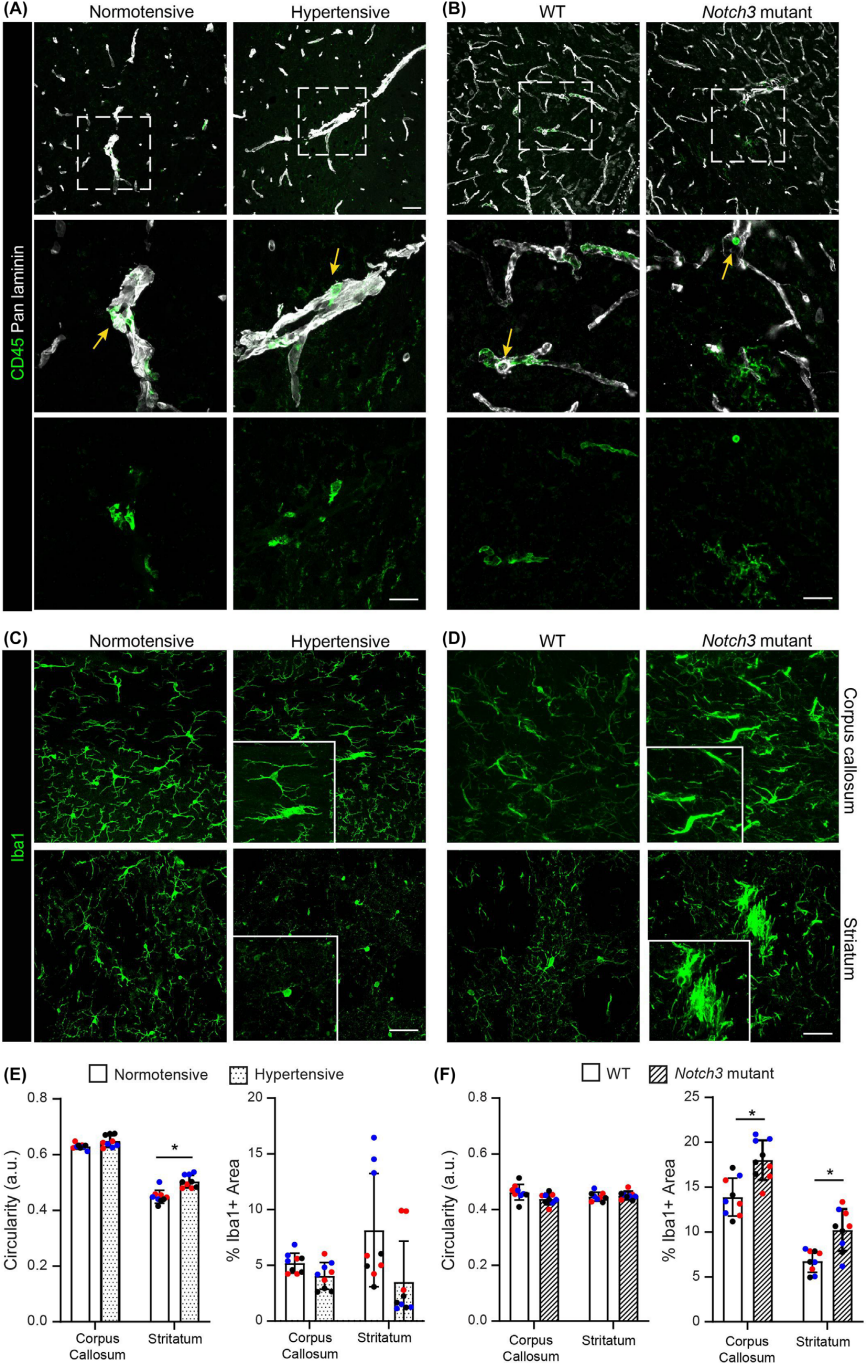
Immune cells in mouse models of SVD. Representative images of double immunofluorescence staining for (A, B) CD45 and pan‐laminin or (C, D) Iba1 alone in (A, C) normotensive and hypertensive BPH/2J and (B, D) WT and *Notch3* mutant brains. The boxed areas in A and B are shown at higher magnifications in the lower panels; arrows mark CD45+ cells within vessels; scale bars in A–D are 50 μm. Quantification of microglial circularity and area occupancy in (E) normotensive and hypertensive BPH/2J and (F) WT and *Notch3* mutant brains; data shown are means ± SDs from 3 to 6 sections/mouse for three mice/genotype; individual mice are colour‐coded. The data were analysed by the Mann–Whitney *U* test; *p < 0.05. a.u. is an arbitrary unit. SVD, small vessel disease; WT, wild‐type.

White matter damage or dysfunction is likely to evoke an inflammatory response. In addition, given the proposed implications of inflammation in SVDs [32, 33, 34], we investigated infiltrating vs resident immune cells in the four SVD models at the ages shown in Table 1. In addition to morphology, CD45^high^Iba1^neg^TMEM119^neg^ immunoreactivity was used to identify infiltrating immune cells, while microglia were identified as CD45^low^Iba1^pos^P2RY12^pos^TMEM119^pos^; co‐staining with a pan‐laminin antibody permitted the identification of blood vessel borders. Regardless of the stage examined, immunofluorescence staining revealed no extravascular CD45^high^ cells in any of the four SVD models (Figure 2A; Figure S5). Isolated CD45^high^ immune cells were detected within blood vessel lumens or vessel walls as defined by pan‐laminin staining (Figure 2A); based on morphology and localisation, these cells probably represent perivascular macrophages. In some samples, the CD45 and Iba1 staining intensities (post thresholding using Otsu or Triangle algorithms) of microglia were elevated compared to those of the controls (Figure 2B), suggesting microglia activation. This was particularly clear in the striatum of hypertensive BPH and *Notch3* mutant mice (Figure 2C,D) associated with white matter changes (Figure 1). Staining for the microglial‐specific markers P2RY12 and TMEM119 (Figure S7), together with enhanced Iba‐1 staining intensity, increased circularity (amoeboid morphology) and reduced area occupancy of individual microglia (due to reduced branching), further supported an activated microglia phenotype (Figure 2C–F). In the case of the *Notch3* mutant, increased numbers of Iba1^+^ cells were apparent, of which high proportions were Iba1^high^ and, hence, activated (Figure 2D). In *Notch3* mutants and hypertensive BPH/2J, activated microglia were also present in areas with myelin changes. In the *Col4a1* mutant, activated microglia were restricted to the sites of haemorrhages (Figure S3B). *Htra1* ^−/−^ mice did not exhibit microglial activation (Figure S4C–D).

### Rare focal sites of extravascular IgG and fibrinogen correlate with sites of delocalised aquaporin‐4

**FIGURE 3:**
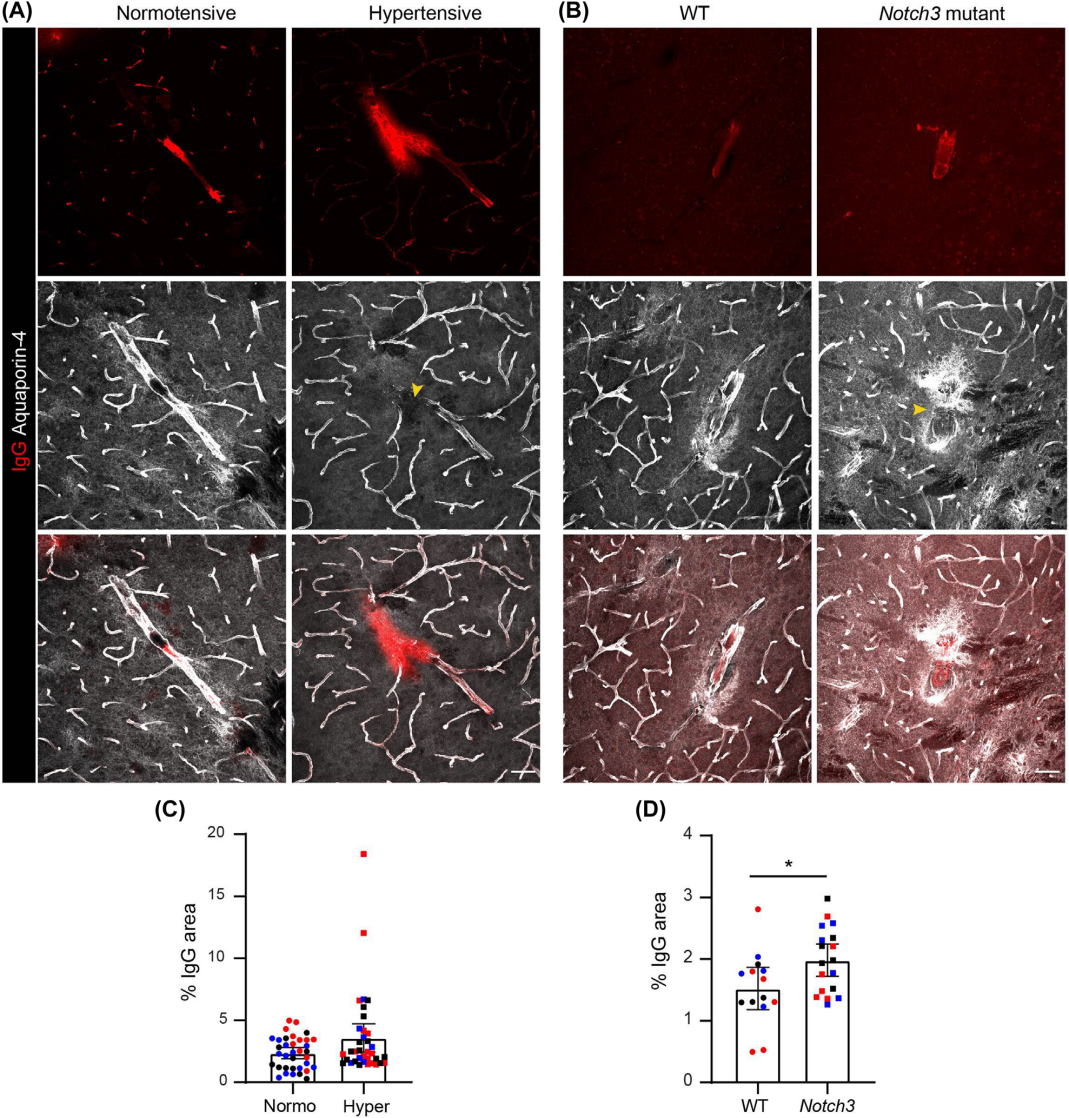
Focal extravascular IgG immunostaining in mouse models of SVD. Double immunofluorescence staining for IgG and aquaporin‐4 in (A) normotensive and hypertensive BPH/2J and (B) WT and *Notch3* mutant brains; scale bars are 50 μm. Arrowheads indicate the absence or aberrant aquaporin‐4 staining. (C, D) Corresponding quantification of the IgG‐positive area; data shown are means ± SDs from 3 to 6 sections/mouse for three mice/genotype; individual mice are colour‐coded. The data were analysed by the Mann–Whitney *U* test; *p < 0.05. SVD, small vessel disease; WT, wild‐type.

BBB dysfunction has long been implicated in the pathophysiology of human SVD [35, 36, 37, 38, 39]. However, the nature of cerebral blood vessel dysfunction and the extent of BBB leakage in SVD are not clear. We, therefore, performed immunofluorescence staining for the plasma proteins IgG and fibrinogen in the brain parenchyma of the SVD mouse strains at the stages outlined in Table 1; co‐staining for aquaporin‐4 or pan‐laminin permitted the identification of vessel borders and hence the assessment of extravascular IgG/fibrinogen. The two proteins differ in molecular weight and, therefore, may indicate different levels of BBB dysfunction. However, their staining patterns mostly colocalised (fibrinogen staining is shown in Figure S6). In all SVD models except *Htra1* ^−/−^, rare focal sites of extravascular IgG were detected already by 6 months (Figure 3A,C; Figure S3B,C; Figure S6). Immunofluorescence staining for aquaporin‐4, which marks the astrocytic endfeet, revealed loss of staining at the sites of extravascular IgG/fibrinogen staining (Figure 3A,C; Figure S3C). However, no correlation was found between areas showing changes in myelination and detection of extravascular IgG or fibrinogen in hypertensive BPH/2J and *Notch3* mutant mice.

Taken together, our data suggest the absence of peripheral immune cell infiltration in all the mouse SVD models examined. Rather, general and widespread microglial activation was apparent with rare, focal sites of extravascular IgG/fibrinogen staining. Only in hypertensive BPH mice and *Notch3* mutants was there evidence of some correlation between sites of microglial changes and myelination defects.

### Validation of mouse data in human SVD autopsy samples

**FIGURE 4:**
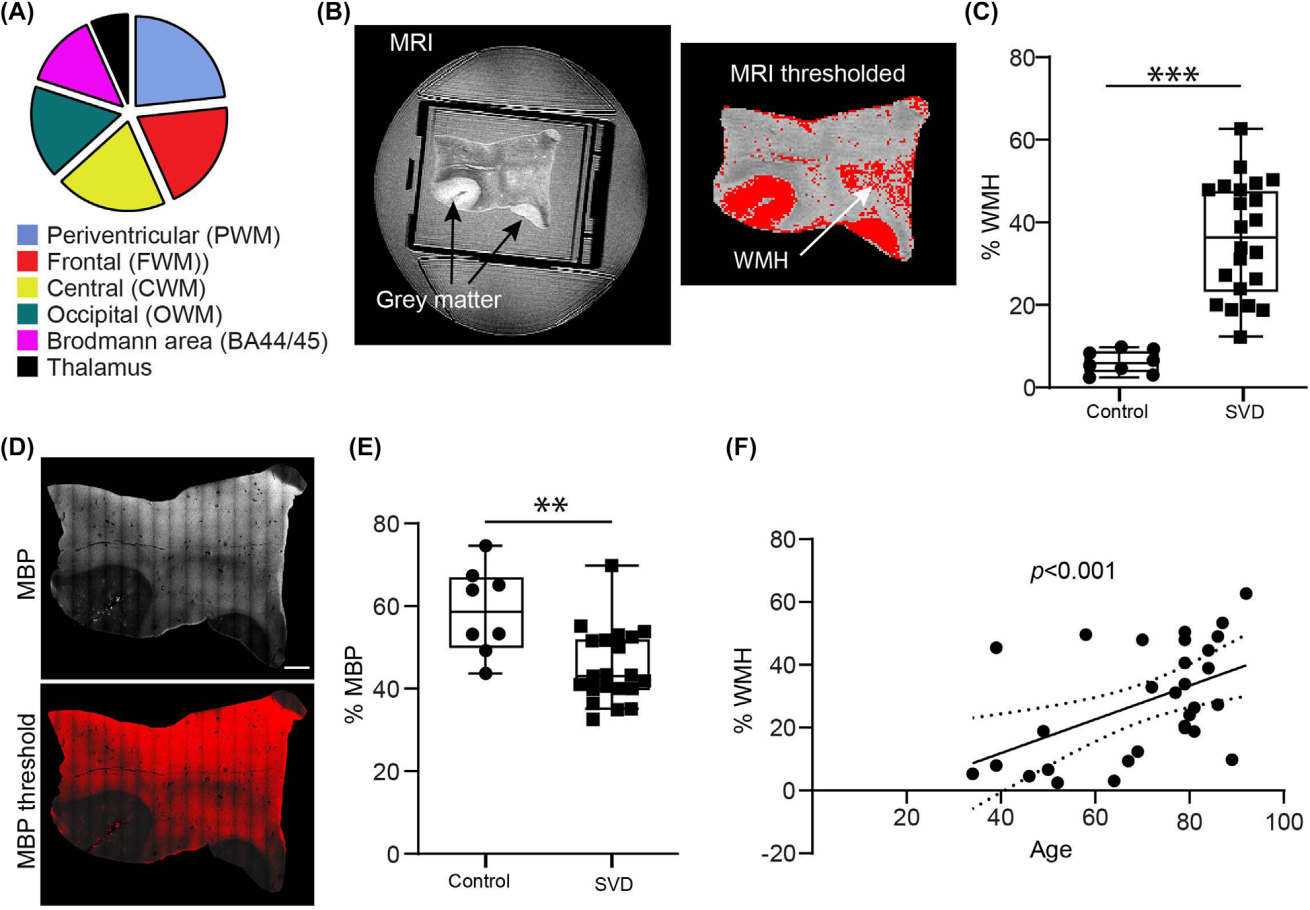
Classification of human autopsy specimens. (A) Pie chart showing the proportions of different white matter regions in the specimens analysed; *n* = 30. (B) Representative postmortem MR image (T2) of a human brain specimen and corresponding thresholded image from which areas of white matter hyperintensity (WMH) were defined. (C) Quantification of the % areas showing WMH. Specimens showing more than 10% WMH were classified as SVD. (D) Immunofluorescence staining for myelin basic protein (MBP) and corresponding thresholded image of the same specimen as in A; scale bar is 2000 μm. (D) Percent area occupied by MBP immunostaining in control and SVD cases. Mann–Whitney test, **p < 0.01, ***p < 0.001. (E) Correlation between age and WMH burden; Pearson correlation, r = 0.4973; **p < 0.01. The dotted line represents the 95% confidence interval. MR, magnetic resonance; SVD, small vessel disease.

To assess whether the changes observed in mouse models of SVD are relevant to human diseases, postmortem SVD brain specimens were examined from different regions of the brain, including the periventricular, frontal, central and occipital white matter (Figure 4A). All samples were sporadic cases of SVD; region‐matched and, where possible, age‐matched controls were also analysed. The WMH burden in each specimen was quantified by thresholding the T2‐weighted images (Figure 4B) [11]. Based on the proportion of WMH per section area (expressed as % WMH), the specimens were classified as controls or SVD cases (Figure 4C). This classification was consistent with the histopathological assessment of the specimens. Each sample was immunofluorescently stained for MBP (Figure 4D), and the extent of staining per section area was determined (expressed as % MBP), revealing less extensive MBP staining in the SVD cases than in the controls (Figure 4E), validating the MRI‐based classification. WMH burden increased with age (*r* = 0.4973, *p* = 0.0052) (Figure 4F).

### Assessment of peripheral immune cells in human SVD samples

**FIGURE 5:**
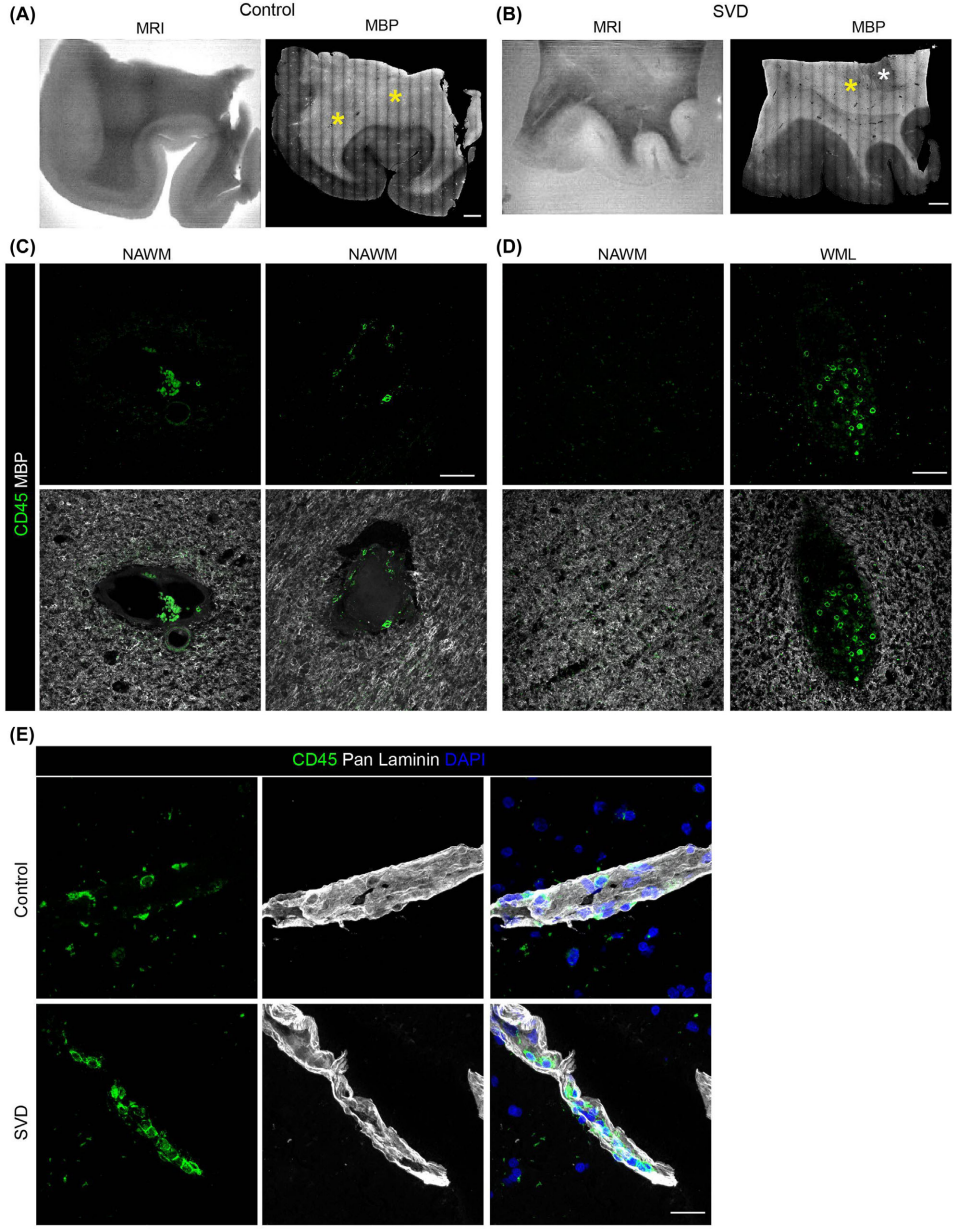
MRI‐guided immune cell localisation. Postmortem (A) MRI (T2) and (B) MBP immunofluorescence staining in control and SVD samples; white asterisks indicate white matter lesions (WMLs), and yellow asterisks indicate normal‐appearing white matter (NAWM). Representative images for immunofluorescence staining of (C, D) CD45 and MBP in the WML and NAWM regions or (E) CD45, pan‐laminin and DAPI to mark all nuclei in SVD vs control samples; scale bars are 1000 μm in A and B and 50 μm otherwise. MBP, myelin basic protein; MRI, magnetic resonance imaging; SVD, small vessel disease.

In human SVD samples, immunofluorescence staining for MBP was correlated with WMH areas according to the MR images (Figure 5A,B). Staining of consecutive sections for CD45 revealed some immune cells within vessels but little or no staining within the brain parenchyma, apart from low‐level staining of microglia; there was no correlation between the extent or intensity of CD45 staining and areas of demyelination, as determined by MBP immunostaining (Figure 5C,D). Corresponding frozen samples from the contralateral hemisphere of the same brain revealed that CD45‐positive cells were detected only within the borders of blood vessels, as defined by pan‐laminin staining (Figure 5E), indicating the absence of immune cell extravasation.

### Focal extravascular fibrinogen in human SVD samples

**FIGURE 6:**
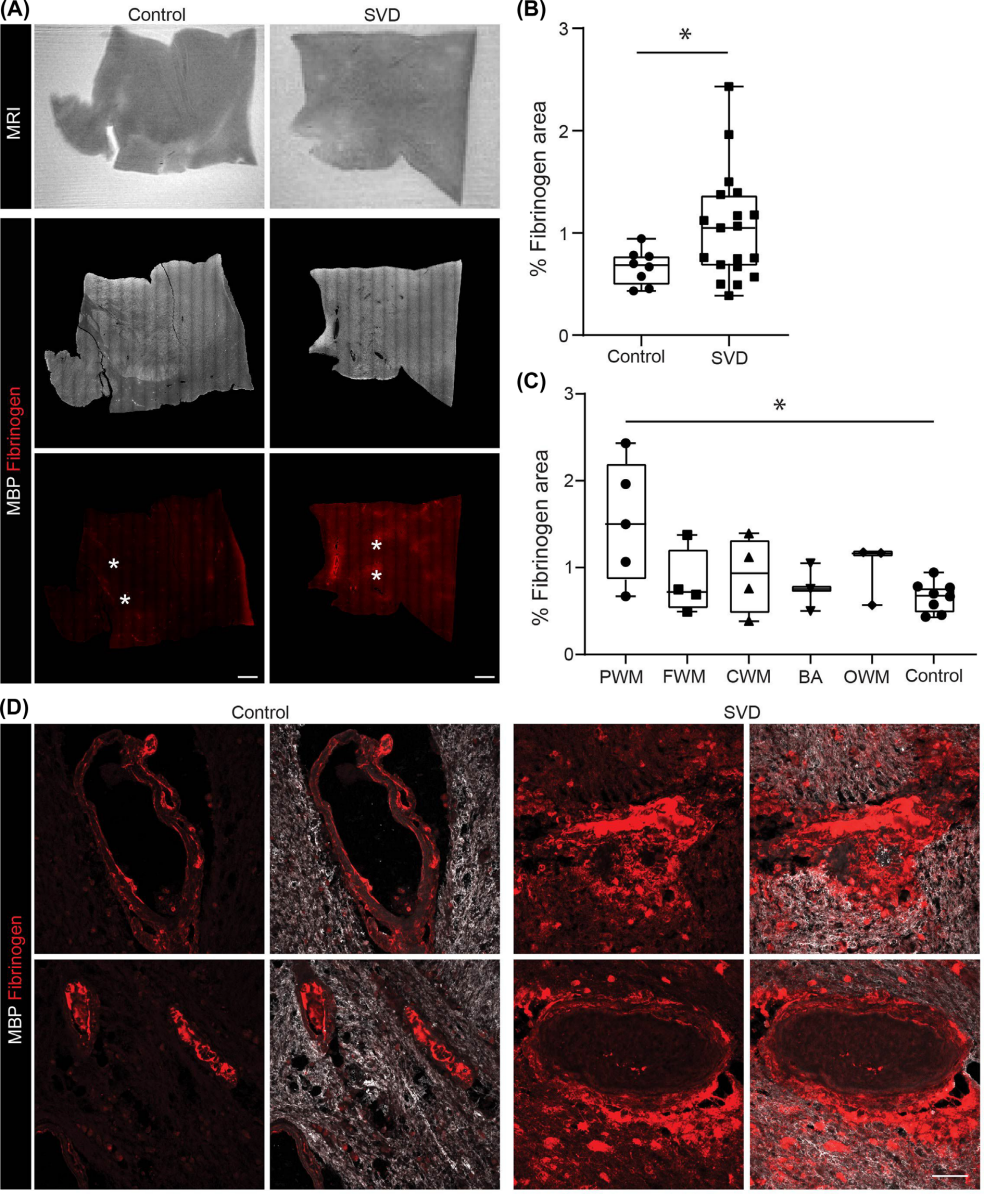
Fibrinogen immunoreactivity in autopsy specimens. (A) Postmortem MRI (T2) and double immunofluorescence staining for MBP and fibrinogen in control and SVD samples. High‐magnification images of the regions marked with asterisks are shown in D. (B) Corresponding quantification of the % area showing fibrinogen immunostaining in SVD and control samples. (C) % Fibrinogen‐positive area in different regions of the brain in SVD samples and controls (periventricular white matter [PWM], frontal white matter [FWM], central white matter [CWM], occipital white matter, Brodmann area 44/45 [BA44/45]). (D) High‐magnification images of double immunofluorescence staining for fibrinogen and MBP in control and SDV samples. The data in B and C are the medians and interquartile ranges for all samples (); the data were analysed using Student's *t‐*test in B and one‐way ANOVA in C, *p < 0.05. The scale bars in A are 1000 μm; otherwise, they are 50 μm. ANOVA, analysis of variance; MBP, myelin basic protein; SVD, small vessel disease;

Immunofluorescence staining and comparison with MRI revealed a greater fibrinogen staining intensity throughout the brain parenchyma in all human SVD samples than in controls, where staining occurred only in vessel walls (Figure 6A–D). Rare sites of focal perivascular staining were detected surrounding larger vessels, which did not, however, consistently correlate with sites of demyelination or WMH (Figure 6A,B) and occurred mainly in periventricular white matter (PWM) regions (Figure 6C). Because staining of formalin‐fixed paraffin sections can result in nonspecific binding of antibodies, we also examined frozen specimens from the contralateral hemisphere of the same patients using three different fibrinogen antibodies. We used pan‐laminin antibody to mark vessel borders and α‐smooth muscle actin to mark arterioles. Using these markers, fibrinogen stainings around vessels of different types and sizes were analysed. Focal perivascular fibrinogen immunoreactivity was confirmed mainly around vessels 15–30 μm in diameter (Figure S8A–B). Approximately one‐third of these vessels were α‐smooth muscle actin positive. Immunofluorescence staining of consecutive sections revealed that all vessels showed normal staining for claudin 5, occludin and caveolin 1 (Figure S9A–B), suggesting some change in vessel permeability but no overt or very subtle changes in junctional or transport molecules. The vessel diameters and α‐smooth muscle actin immunoreactivity suggested that the fibrinogen‐positive vessels were small arterioles or postcapillary venules.

### Microglia in WMLs have a circular morphology and increased MBP burden

**FIGURE 7:**
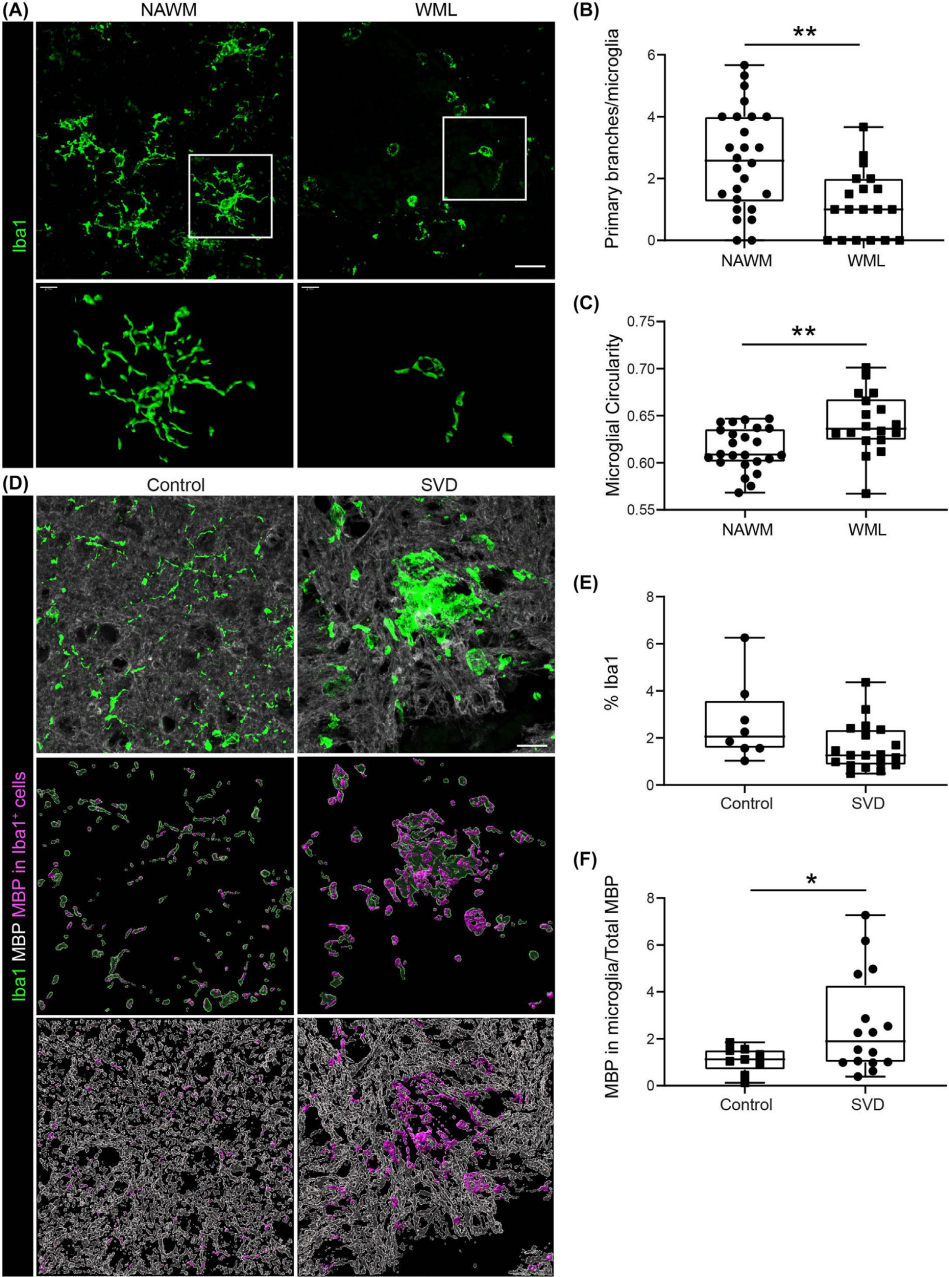
Microglial reactivity in white matter lesions (WMLs). (A) Immunofluorescence staining of Iba1 + microglia in the NAWM and WML regions of the same specimen; the lower panels show 3D reconstructions of the boxed areas; scale bars are 50 μm. Corresponding quantification of (B) primary microglial branches (26 NAWM and 19 WML) and (C) microglial circularity (24 NAWM and 18 WML); the data shown are medians with interquartile ranges and were analysed using the Mann–Whitney test for primary microglial branches and Welch's *t* test for microglial circularity; *p < 0.05, **p < 0.01. (D) Double immunofluorescence staining for MBP and Iba1 in control and SVD specimens. The lower panels show 3D reconstructions. MBP inside microglia is pseudo‐coloured in magenta. The scale bar is 50 μm. (E) Quantification of Iba‐1‐positive area (%) in control and WML samples. (F) MBP inside Iba1 + microglia expressed as a % of the total MBP area in control and WML samples. The data in E and F are the medians with interquartile ranges and were analysed using Student's *t*‐test (control = 8, SVD = 19 samples); *p < 0.01. MBP, myelin basic protein; NAWM, normal‐appearing white matter.

As in the mouse SVD model samples, microglia marked by Iba1 immunostaining exhibited altered morphology in WMLs. To avoid confounding factors such as age, postmortem interval and tissue fixation time, microglia in WMHs and normal white matter regions from the same specimen were compared (Figure 7A). In all 30 samples examined, microglia in the MRI‐defined WMLs had fewer branches than those in normal white matter regions and greater circularity (Figure 7A–C), suggesting an activated phenotype. In WMHs, double immunofluorescence staining for Iba1 and MBP revealed low myelin staining, suggesting the phagocytosis of myelin by microglia (Figure 7D), a further indication of an activated phenotype. The total area occupied by microglia in WMLs was not significantly different from that in NAWMs, although there was a tendency toward a reduction in the area occupied by microglia in WMHs (Figure 7E). Moreover, WMLs had lower MBP staining. Therefore, MBP inside microglia was normalised to the total MBP in each section. 3D reconstruction of Iba1 and MBP immunostaining suggested that the amount of phagocytosed MBP was greater in WMLs than in NAWMs, with a slightly reduced microglial area, probably due to their circular morphology and reduced branching (Figure 7F).

## DISCUSSION

This study assessed immune cell localisation and activation status in hypertensive BPH mice *Col4a1* mutant, *Notch3* mutant and *Htra1* ^*−/−*^ mouse models of SVD and in human autopsy specimens. Using MRI and immunostaining for MBP, WMH sites were identified and shown to lack CD45^high^ immune cell infiltration at any stage of lesion development. Rather, microglial activation, as defined by morphological changes in cell shape and high intensity of Iba1 staining, was a common feature of both mouse and human SVD samples. The presence of rare focal sites of extravascular serum proteins surrounding small arterioles or venules was a further characteristic of both mouse and human SVD but was not restricted to WMHs.

Immunofluorescent staining and histology revealed that only BPH/2J hypertensive mice and *Notch3* mutant mice (Notch3R169G/CADASIL) exhibited clear white matter changes, as indicated by reduced FA values and areas of MBP immunostaining. *Col4a1* mutants share several features with hypertensive and *Notch3* mutant mice, including microglial activation, but *Htra1* ^−/−^ mice are different. Although *Htra1* mutations have been identified in a small subset of SVD patients (CARASIL patients), complete elimination in the knockout mouse has only a subtle phenotype, suggesting that other compensatory mechanisms do not occur when there are specific mutations in patients or that this protein has a different function in rodents than in humans. This may be the reason that the pattern of results observed in the other 3 SVD mouse models was not detectable in *Htra1^−/−^* mice.

Hypertensive BPH 2J mice have not been extensively characterised for white matter changes and in this study were shown to have a reduced area of MBP immunoreactivity which correlated with reduced FA values in the periventricular striatum. Similar results have been reported in spontaneously hypertensive stroke‐prone rats [40]. Although myelin degradation in the corpus callosum of *Notch3* mutant mice has been previously reported [41, 42], we showed here that reduced MBP staining also occurs in the periventricular striatum, a feature that has been frequently observed in MRI scans of human SVD patients, including CADASIL patients [43, 44]. Neither the *Col4a1* mutant (6 months) nor *Htra1* ^−/−^ mice showed substantial changes in MBP staining (6 and 12 months). Although white matter changes have not been extensively characterised in *Htra1* ^*−/−*^ mice, our results are consistent with published data reporting only some disorganisation of myelin in 26‐month‐old *Htra1* ^*−/−*^ mice [45]. *Col4a1* mutant mice exhibit focal microhaemorrhages and macrohaemorrhages in the interbrain region [28]. Indeed, MRI scans of the *Col4a1* mutant mice revealed hypointense signals in the interbrain regions, indicating haemorrhage. Taken together, these findings suggest that these mouse models do not exhibit substantial white matter changes despite vascular pathology.

The absence of extravascular immunofluorescent staining for CD45^high^ cells in all four mouse models and human SVD samples suggested that leukocyte extravasation is not a common feature of SVD. We did not observe the presence of CD45^high^ infiltrating immune cells in the brain parenchyma except at specific sites of haemorrhage in *Col4a1* mutant mice. In hypertensive BPH/2J and *Notch3* mutant mice, microglial morphology was altered in areas showing reduced MPB staining and MRI changes. These results are in accordance with a recent report of microglial activation in BPH/2J mice that was reversed by amlodipine treatment and blood pressure reduction [27] and by studies on *Notch3* mutant and *Col4a1* mutant mice [17, 29]. Generally, increased intensity of the Iba‐1 immunostaining has been regarded as an indication of microglial activation. In addition, we highlight the reduced branching and circular morphology of the activated microglia.

In addition to microglial activation and myelin damage, isolated instances of extravascular IgG/fibrinogen were observed in all mouse models except for *Htra1* ^−/−^ mice and were associated with the loss of aquaporin‐4 staining at astrocyte endfeet. Serum proteins normally do not penetrate the BBB, and their presence in the brain parenchyma suggests a focal change in the neurovascular unit which would also occur in the case of microhaemorrhages, a hallmark of SVD. Similarly, a reduction in aquaporin‐4 expression or its delocalisation from astrocyte endfeet [46] has been reported in ischaemic stroke where BBB function is compromised [47]. Our data, therefore, are consistent with focal changes in vessel permeability, which has been proposed by others using intravenously applied exogenous tracers [27, 28, 41] but has been a controversial issue in *Notch3* mutants. Additionally, human studies have suggested both generalised BBB dysfunction and ‘hot spots’ where lesions are forming or tissue is disintegrating [37].

The Htra1^−/−^ mouse model (CARASIL) lacked any substantial pathology and was different from other mouse models. The three major features of other 3 SVD mouse models—a lack of CD45^high^ immune cell infiltration, broad microglial activation and occasional focal sites of extravascular fibrinogen/IgG—were confirmed in autopsy human nonamyloid SVD specimens from the periventricular, central, frontal and occipital white matter regions, in which WMHs were identified using postmortem MRI and immunostaining. The SVD sample cohort employed represents a broader range of white matter regions compared to published studies that focused on either the periventricular or deep white matter [30, 36, 37]. In our study, regions of white matter lesions (WMLs) were identified by unbiased thresholding of T2‐MR images, which were then correlated with thresholded tile scans of MBP‐stained sections. This revealed that SVD cases had at least 10% or more WML area on T2 scans and had a significantly lower percentage of MBP staining. Our comparisons included not only WMLs and controls but also WMLs and normal‐appearing white matter (NAWM) within the same specimen. This strategy overcomes the confounding factors and is consistent with the published literature [48].

CD45^high^ peripheral immune cells in human SVD samples did not colocalise with the reduced MBP in either WMLs or NAWMs, indicating their absence in the parenchyma in general. Moreover, immunostaining of corresponding frozen specimens revealed immune cells only within the borders of pan‐laminin staining, which includes the astroglial basement membrane that marks the border to the CNS parenchyma [49, 50], excluding the possibility of immune cell infiltration from the circulation. As in mouse SVD models, the microglia in the WML exhibited greater circularity and ameboid morphology and the presence of MBP within Iba1^+^ microglia, suggesting enhanced phagocytotic behaviour [51], consistent with an activated phenotype. This suggests that previous reports of neuroinflammation in SVD [48] are likely due to the detection of activated resident microglia rather than infiltrating immune cells, possibly secondary to myelin disruption.

Although focal sites of extravascular fibrinogen surrounding small vessels were also detected in human SVD samples, there was no spatial colocalisation with activated microglia. This finding is consistent with a previous cross‐sectional positron emission tomography imaging study of SVD patients, which revealed that focal sites of BBB leakage were distinct from sites of microglial activation [39]. However, it is not known whether microglial activation occurs prior to or as a consequence of BBB changes. The broad activation of microglia in mouse and human SVD brains, which correlated with changes in MBP staining but not sites of extravascular fibrinogen staining, is, therefore, more likely to reflect the response to myelin damage. Our data further support the concept that subtle changes in vessel permeability, for example, to serum proteins, vs immune cells are molecularly distinct processes, as shown elegantly in mouse studies [52]. Furthermore, we highlight the presence of serum proteins around noncapillary vessels like small arterioles and venules. The vessel type identity (capillary, artery or vein) was based on not only its diameter but also on the presence or absence of smooth muscle actin. We used the basement membrane markers as well as endothelial markers for blood vessel characterisation. It is important to note that our samples primarily comprised nonamyloid SVD cases. Earlier studies by Magaki et al essentially dealt with cerebral amyloid angiopathy cases [53]. Their study showed increased fibrinogen in capillaries, while our data indicate increased fibrinogen around noncapillary vessels and mainly in the parenchyma instead of vessel lumen.

To the best of our knowledge, our study is the first to comprehensively compare several mouse SVD models with a broad range of human SVD samples. We systematically evaluated the nature of neuroinflammation in SVD and showed that neuroinflammation manifests as the activation of resident immune cells rather than the infiltration of peripheral immune cells. This finding has important implications for therapies targeting peripheral inflammation in vascular dementia and stroke caused by SVDs.

### Limitations statement

Microhaemorrhagic lesions as a feature of SVD were not directly investigated. Rather, fibrinogen staining was employed to identify changes in vessel permeability. The study used CD45 as a pan immune cell marker and Iba1, TMEM119 as microglia marker while no specific markers were used for immune cell types like neutrophils, lymphocytes and macrophages. As a third of the vessels showing extravascular fibrinogen staining analysed in this study were smooth muscle actin positive, this may be an indication of arteriole lesions as an additional SVD feature which, however, requires further analyses. A further limitation of our study was the criteria employed for the classification of specimens into SVD and non‐SVD cases, which was based on the WMH criterion from T2 imaging and MBP immunostaining (which matched with the neuropathological assessment from the brain bank). We acknowledge that these are ex vivo assessments and do not necessarily represent MRI in living patients.

## AUTHOR CONTRIBUTIONS

Lydia Sorokin designed the study and supervised the experiments. Tushar Deshpande, Melanie‐Jane Hannocks, Kishan Kapupara, Sai Kiran Reddy Samawar performed the experiments. Lydia Wachsmuth and Cornelius Faber did the mouse brain MRI experiments. Colin Smith did the human post mortem MRI and helped in data interpretation and discussion. Joanna Wardlaw helped in data interpretation and discussion. Tushar Deshpande analysed the data. Lydia Sorokin and Tushar Deshpande wrote the manuscript. All the authors read and corrected the manuscript.

## CONFLICT OF INTEREST STATEMENT

CS is an executive editor of Neuropathology and Applied Neurobiology. The Editors of Neuropathology and Applied Neurobiology are committed to peer‐review integrity and upholding the highest standards of review. As such, this article was peer‐reviewed by independent, anonymous expert referees, and the authors (including CS) had no role in either the editorial decision or the handling of the paper.

### PEER REVIEW

The peer review history for this article is available at https://www.webofscience.com/api/gateway/wos/peer-review/10.1111/nan.13015.

## ETHICS STATEMENT

Samples from mouse models were obtained as per local Animal Welfare guidelines. Autopsy brain specimens were obtained from the Edinburgh Brain Bank. Ethical approval is not required for publishing this work.

## Supporting information


**Figure S1:** **Regions of interest for quantifying fractional anisotropy (FA) maps in fixed mouse brains.** 3D reconstruction of the mouse brain using Brain Explorer 2 (version 2.3.5 from the Allen Institute of Brain Sciences) showing approximate locations of the FA map sections. Ten 200 μm thick sections from the anterior region of the brain were quantified for FA. The regions of interest analysed were the corpus callosum and striatum.
**Figure S2: White matter integrity in *the Col4a1* mutant.** (A) MRI of fixed WT and *Col4a1* mutant brains. The upper panels show T2 maps; the lower panels are fractional anisotropy (FA) maps; the scale bar is 1 mm. (B) Quantification of MR images; the X‐axis represents the number of dorsal to ventral slices. The upper graphs show the average FA values ± 95% confidence intervals in the corpus callosum and striatum; the lower graphs show the % areas above the threshold in the FA maps. (C) Myelin basic protein (MBP) immunofluorescence staining of the corpus callosum and striatum of the WT and *Col4a1* mutant brains; the scale bar represents 50 μm. (D) Corresponding quantification of MBP immunostaining; data shown are means ± SDs of 9 sections from 3 mice per genotype; individual mice are colour‐coded.
**Figure S3: Haemorrhages in the *Col4a1* mutant.** (A) MRI of fixed WT and *Col4a1* mutant brains. The T2 map shows the hypointense spots in the hippocampus and interbrain region of the *Col4a1* mutant; the scale bar is 1 mm. A magnified image of the boxed areas is shown to the right; the scale bar is 0.5 mm. Double immunofluorescence staining of WT and *Col4a1* mutant brain sections in the interbrain for (B) IgG and Iba1 or (C) IgG and aquaporin‐4; arrowheads in C mark the loss of aquaporin‐4 staining in the regions of extravascular IgG. The scale bars in B and C are 50 μm.
**Figure S4: *Htra1*** ^***‐‐−***^ ***(*CARASIL) mice** *.* (A) Myelin basic protein (MBP) immunofluorescence staining of the corpus callosum and striatum of WT and *Htra1* ^*−/−*^ brain sections. (B) Corresponding quantification of MBP immunostaining; the data shown are the mean percentage of MBP‐positive area ± SD for 9 sections from 3 mice/genotype. The data were analysed by Student's t‐test. (C) Iba1 immunofluorescence staining of the corpus callosum and striatum of WT and *Htra1* ^*−/−*^ mice. (D) Corresponding quantification of the % Iba1 + area and microglial circularity (a.u. is arbitrary); the data are presented as the mean percentage of Iba1 + area ± SD for 9 sections from 3 mice/genotype, individual mice are colour‐coded. The data were analysed by the Mann‐Whitney U test. The scale bars in A and C are 50 μm.
**Figure S5: CD45 positive immune cells in *Col4a1* mutant and *Htra1*** ^***‐‐−***^
CD45 (green)‐PECAM‐1 (magenta) immunostaining in *Col4a1* mutant and *Htra1* ^*‐‐−*^ mouse brains. CD45‐positive immune cells reside within the PECAM‐1 positive blood vessels. The scale bar in the upper panel is 50 μm. Scale bars in the enlarged images are 25 μm.
**Figure S6: Fibrinogen immunoreactivity in mouse models of SVD.** Normotensive and hypertensive mouse brain sections and WT and *Notch3* mutant brain sections were double immunofluorescently stained for fibrinogen and pan‐laminin; boxed areas are shown at higher magnification in the lower panels; scale bars are 50 μm in the upper panels and 25 μm in the lower 2 panels.
**Figure S7: Microglia‐specific markers in hypertensive and *Notch3* mutant mice.** Iba1 (white) and TMEM119 (red) immunostaining in hypertensive and *Notch3* mutant brains showing colocalisation of these markers. Scale bars are 50 μm.
**Figure S8: Fibrinogen staining is associated with arterioles and venules.** (A) Overview images showing double immunofluorescence staining for pan‐laminin and fibrinogen in control and SVD specimens and (B) high magnifications of the boxed regions in A. (C) Histogram showing the percent frequency (% of total vessels analysed) of vessel diameters associated with extravascular fibrinogen staining; the data are the means from 20 different samples. (D) Representative α‐smooth muscle actin (SMA) and fibrinogen double immunofluorescence staining of an SVD sample. (E) Pie chart showing the proportion of α‐SMA‐positive vessels associated with extravascular fibrinogen staining (76 vessels from 13 specimens). Scale bars in A are 1000 μm and 50 μm in B and D.
**Figure S9: Tight junctions and caveolae proteins in human brain samples.** (A) Fibrinogen‐Claudin‐5 immunostaining showing normal appearing (upper panel) and dysfunctional blood vessels (lower panel). Scale bar 10 μm. (B) Fibrinogen‐occludin immunostaining showing normal appearing (upper panel) and dysfunctional blood vessels (lower panel). Scale bar 10 μm. (C) Fibrinogen‐Caveolin‐1 immunostaining showing normal appearing (upper panel) and dysfunctional blood vessels (lower panel). Scale bar 10 μm.

Supplementary Figures

## Data Availability

The data that support the findings of this study are available from the corresponding author upon reasonable request.
