# Supplementary material for: Microglial activation without peripheral immune cell infiltration characterises mouse and human cerebral small vessel disease
Source: Neuropathol Appl Neurobiol. 2024 Nov 14;50(6):e13015. doi: 10.1111/nan.13015 (PMC11618487; doi:10.1111/nan.13015)
Supplement: Supplementary file 1 — Figure S1: Regions of interest for quantifying fractional anisotropy (FA) maps in fixed mouse brains. 3D reconstruction of the mouse brain using Brain Explorer 2 (version 2.3.5 from the Allen Institute of Brain Sciences) showing approximate locations of the FA map sections. Ten 200 μm thick sections from the anterior region of the brain were quantified for FA. The regions of interest analysed were the corpus callosum and striatum. Figure S2: White matter integrity in the Col4a1 mutant. (A) MRI of fixed WT and Col4a1 mutant brains. The upper panels show T2 maps; the lower panels are fractional anisotropy (FA) maps; the scale bar is 1 mm. (B) Quantification of MR images; the X‐axis represents the number of dorsal to ventral slices. The upper graphs show the average FA values ± 95% confidence intervals in the corpus callosum and striatum; the lower graphs show the % areas above the threshold in the FA maps. (C) Myelin basic protein (MBP) immunofluorescence staining of the corpus callosum and striatum of the WT and Col4a1 mutant brains; the scale bar represents 50 μm. (D) Corresponding quantification of MBP immunostaining; data shown are means ± SDs of 9 sections from 3 mice per genotype; individual mice are colour‐coded. Figure S3: Haemorrhages in the Col4a1 mutant. (A) MRI of fixed WT and Col4a1 mutant brains. The T2 map shows the hypointense spots in the hippocampus and interbrain region of the Col4a1 mutant; the scale bar is 1 mm. A magnified image of the boxed areas is shown to the right; the scale bar is 0.5 mm. Double immunofluorescence staining of WT and Col4a1 mutant brain sections in the interbrain for (B) IgG and Iba1 or (C) IgG and aquaporin‐4; arrowheads in C mark the loss of aquaporin‐4 staining in the regions of extravascular IgG. The scale bars in B and C are 50 μm. Figure S4: Htra1 ‐‐− (CARASIL) mice . (A) Myelin basic protein (MBP) immunofluorescence staining of the corpus callosum and striatum of WT and Htra1 −/− brain sections. (B) Corresponding quan [file NAN-50-e13015-s002.docx]

**Supplementary Data**

**Figure S1: Regions of interest for quantifying fractional anisotropy (FA) maps in fixed mouse brains.** 3D reconstruction of the mouse brain using Brain Explorer 2 (version 2.3.5 from the Allen Institute of Brain Sciences) showing approximate locations of the FA map sections. Ten 200 µm thick sections from the anterior region of the brain were quantified for FA. The regions of interest analysed were the corpus callosum and striatum.

**Figure S2: White matter integrity in *the Col4a1* mutant.** (A) MRI of fixed WT and *Col4a1* mutant brains. The upper panels show T2 maps; the lower panels are fractional anisotropy (FA) maps; the scale bar is 1 mm. (B) Quantification of MR images; the X-axis represents the number of dorsal to ventral slices. The upper graphs show the average FA values ± 95% confidence intervals in the corpus callosum and striatum; the lower graphs show the % areas above the threshold in the FA maps. (C) Myelin basic protein (MBP) immunofluorescence staining of the corpus callosum and striatum of the WT and *Col4a1* mutant brains; the scale bar represents 50 µm. (D) Corresponding quantification of MBP immunostaining; data shown are means ± SDs of 9 sections from 3 mice per genotype; individual mice are colour-coded.

**Figure S3: Haemorrhages in the *Col4a1* mutant.** (A) MRI of fixed WT and *Col4a1* mutant brains. The T2 map shows the hypointense spots in the hippocampus and interbrain region of the *Col4a1* mutant; the scale bar is 1 mm. A magnified image of the boxed areas is shown to the right; the scale bar is 0.5 mm. Double immunofluorescence staining of WT and *Col4a1* mutant brain sections in the interbrain for (B) IgG and Iba1 or (C) IgG and aquaporin-4; arrowheads in C mark the loss of aquaporin-4 staining in the regions of extravascular IgG. The scale bars in B and C are 50 µm.

**Figure S4: *Htra1^-/-^ (*CARASIL) mice***.* (A) Myelin basic protein (MBP) immunofluorescence staining of the corpus callosum and striatum of WT and *Htra1^-/-^* brain sections. (B) Corresponding quantification of MBP immunostaining; the data shown are the mean percentage of MBP-positive area ± SD for 9 sections from 3 mice/genotype. The data were analysed by Student’s t-test. (C) Iba1 immunofluorescence staining of the corpus callosum and striatum of WT and *Htra1^-/-^* mice. (D) Corresponding quantification of the % Iba1+ area and microglial circularity (a.u. is arbitrary); the data are presented as the mean percentage of Iba1+ area ± SD for 9 sections from 3 mice/genotype, individual mice are colour-coded. The data were analysed by the Mann-Whitney U test. The scale bars in A and C are 50 µm.

**Figure S5: CD45 positive immune cells in *Col4a1* mutant and *Htra1^-/-^***

CD45 (green)-PECAM-1 (magenta) immunostaining in *Col4a1* mutant and *Htra1^-/-^* mouse brains. CD45-positive immune cells reside within the PECAM-1 positive blood vessels. The scale bar in the upper panel is 50 µm. Scale bars in the enlarged images are 25 µm.

**Figure S6: Fibrinogen immunoreactivity in mouse models of SVD.** Normotensive and hypertensive mouse brain sections and WT and *Notch3* mutant brain sections were double immunofluorescently stained for fibrinogen and pan-laminin; boxed areas are shown at higher magnification in the lower panels; scale bars are 50 µm in the upper panels and 25 µm in the lower 2 panels.

**Figure S7: Microglia-specific markers in hypertensive and *Notch3* mutant mice**

Iba1 (white) and TMEM119 (red) immunostaining in hypertensive and *Notch3* mutant brains showing colocalisation of these markers. Scale bars are 50 µm.

**Figure S8: Fibrinogen staining is associated with arterioles and venules.** (A) Overview images showing double immunofluorescence staining for pan-laminin and fibrinogen in control and SVD specimens and (B) high magnifications of the boxed regions in A. (C) Histogram showing the percent frequency (% of total vessels analysed) of vessel diameters associated with extravascular fibrinogen staining; the data are the means from 20 different samples. (D) Representative α-smooth muscle actin (SMA) and fibrinogen double immunofluorescence staining of an SVD sample. (E) Pie chart showing the proportion of α-SMA-positive vessels associated with extravascular fibrinogen staining (76 vessels from 13 specimens). Scale bars in A are 1000 µm and 50 µm in B and D.

**Figure S9: Tight junctions and caveolae proteins in human brain samples**

(A) Fibrinogen-Claudin-5 immunostaining showing normal appearing (upper panel) and dysfunctional blood vessels (lower panel). Scale bar 10 µm.

(B) Fibrinogen-occludin immunostaining showing normal appearing (upper panel) and dysfunctional blood vessels (lower panel). Scale bar 10 µm.

(C) Fibrinogen-Caveolin-1 immunostaining showing normal appearing (upper panel) and dysfunctional blood vessels (lower panel). Scale bar 10 µm.

**Table 3:** Postmortem Specimens Analysed (**BOLD** Marked Controls)

| **ID** | **Sex** | **Age** | ***PMI (h)** | **Cause of Death/**  **Primary (1) & Secondary (2) Pathology** | **§BBN** | **Region** |
| --- | --- | --- | --- | --- | --- | --- |
| SD052/15 | M | 84 | 83 | Intracerebral haemorrhage | BBN001.28405 | PWM |
| SD012/16 | M | 81 | 77 | (1) Aspiration pneumonia  (2) Intracerebral haemorrhage | BBN001.28789 | PWM |
| *SD037/16* | F | 84 | *105* | (1) Ischaemic heart disease complications and bilateral lower leg fractures. (2) Previous stroke, (3) Severe small vessel disease | BBN001.29465 | PWM |
| SD001/16 | M | 79 | 72 | (1) Suspected lung carcinoma. (2) Pneumonia | BBN001.28406 | PWM |
| SD003/16 | M | 70 | 82 | (1) Aspiration pneumonia. (2) Intracerebral haemorrhage | BBN001.28408 | PWM |
| ***SD008/18*** | **M** | **52** | ***22*** | **(1) Pulmonary thromboembolism. (2) Deep vein thrombosis** | **BBN001. 2427** | **PWM** |
| ***SD038/16*** | **M** | **39** | ***76*** | **Accidental death** | **BBN001.29466** | **PWM** |
| SD024/17 | M | 72 | 60 | (1) Myocardial infarction, (2) Coronary artery atheroma | BBN001.30178 | FWM |
| SD025/15 | M | 77 | 122 | (1) Aspiration pneumonia, (2) Right intracerebral haemorrhage | BBN001.26496 | FWM |
| SD037/15 | F | 81 | 59 | (1) Acute left-sided intracerebral haemorrhage, (2) Atrial fibrillation | BBN001.26499 | FWM |
| ***SD008/16*** | **M** | **89** | ***21*** | **Intracerebral haemorrhage** | **BBN001.28416** | **FWM** |
| SD034/16 | F | 79 | 68 | (1) Intracerebral haemorrhage. (2) Hypertension | BBN001.29086 | FWM |
| ***SD021/17*** | **M** | **67** | ***68*** | **Ischaemic heart disease** | **BBN001.30147** | **FWM** |
| SD018/16 | F | 79 | 61 | Metastatic breast cancer | BBN001.28794 | CWM |
| SD025/16 | M | 79 | 57 | (1) Chronic lymphocytic leukaemia, (2) Type 2 diabetes, (3) Myelofibrosis, (4) Idiopathic thrombocytopaenia purpura | BBN001.28797 | CWM |
| SD051/15 | M | 79 | 49 | (1) Hospital-acquired pneumonia. (2) Alcohol-related liver cirrhosis with ascites. (3) Severe ^#^LVSD. (4) Chronic obstructive pulmonary disease | BBN001.28402 | CWM |
| ***SD033/16*** | **M** | **46** | ***110*** | **(1) Ischaemic heart disease with coronary artery thrombosis. (2) Coronary artery atherosclerosis** | **BBN001.29085** | **CWM** |
| ***SD032/17*** | **M** | **80** | ***85*** | **(1) Myocardial infarction. (2) Severe aortic stenosis. (3) Advanced Lewy Body dementia** | **BBN001.30854** | **CWM** |
| *SD049/16* | M | 50 | *70* | (1) Ischaemic heart disease. (2) Coronary artery atherosclerosis | BBN001.29540 | CWM |
| SD043/15 | F | 87 | 26 | (1) Right basal ganglia intracerebral haemorrhage | BBN001.26722 | OWM |
| SD028/15 | F | 92 | 50 | Intracerebral haemorrhage | BBN001.26127 | OWM |
| *SD019/16* | M | 86 | *88* | (1) Aspiration pneumonia. (2) Intracerebral haemorrhage. (3) Alzheimer's disease | BBN001.28798 | OWM |
| ***SD009/16*** | **F** | **64** | ***114*** | **Complications of small bowel ischaemia** | **BBN001.28563** | **OWM** |
| SD048/16 | M | 49 | 94 | (1) Ischaemic heart disease. (2) Coronary artery atherosclerosis | BBN001.29531 | OWM |
| SD017/16 | F | 79 | 72 | NA | BBN001.28793 | BA44/45 |
| SD024/16 | F | 86 | 54 | (1) Hospital-acquired pneumonia, (2) Deep left intraparenchymal haemorrhage | BBN001.28800 | BA44/45 |
| SD011/17 | M | 69 | 74 | (1) Complications of ischaemic heart disease and pyelonephritis | BBN001.29881 | BA44/45 |
| ***SD038/17*** | **M** | **34** | ***99*** | **(1) Ischaemic heart disease. (2) Coronary artery atherosclerosis** | **BBN001.30972** | **BA44/45** |
| *SD022/16* | M | 39 | *86* | (1) Ischaemic heart disease, (2) Coronary artery atherosclerosis | BBN001.28959 | Thalamus |
| *SD032/16* | M | 58 | *104* | (1) Ischaemic heart disease. (2) Coronary artery atherosclerosis | BBN001.29084 | Thalamus |

*PMI = Postmortem interval; §BBN = Brain Bank ID, ^#^LVSD = Left ventricular systolic dysfunction.
