# Supplementary material for: Microglial activation without peripheral immune cell infiltration characterises mouse and human cerebral small vessel disease
Source: Neuropathol Appl Neurobiol. 2024 Nov 14;50(6):e13015. doi: 10.1111/nan.13015 (PMC11618487; doi:10.1111/nan.13015)

Supplementary Fig. 1

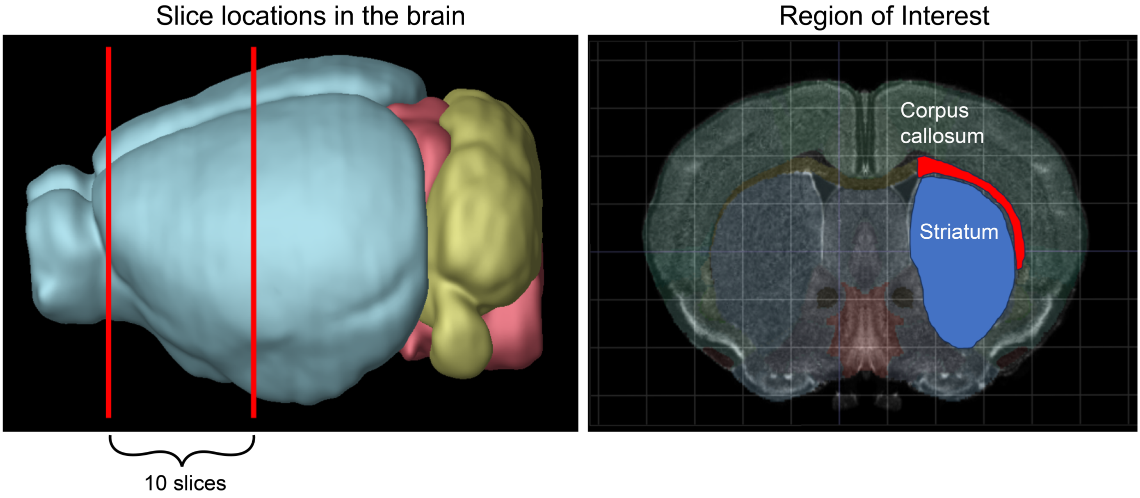

Supplementary Fig. 2

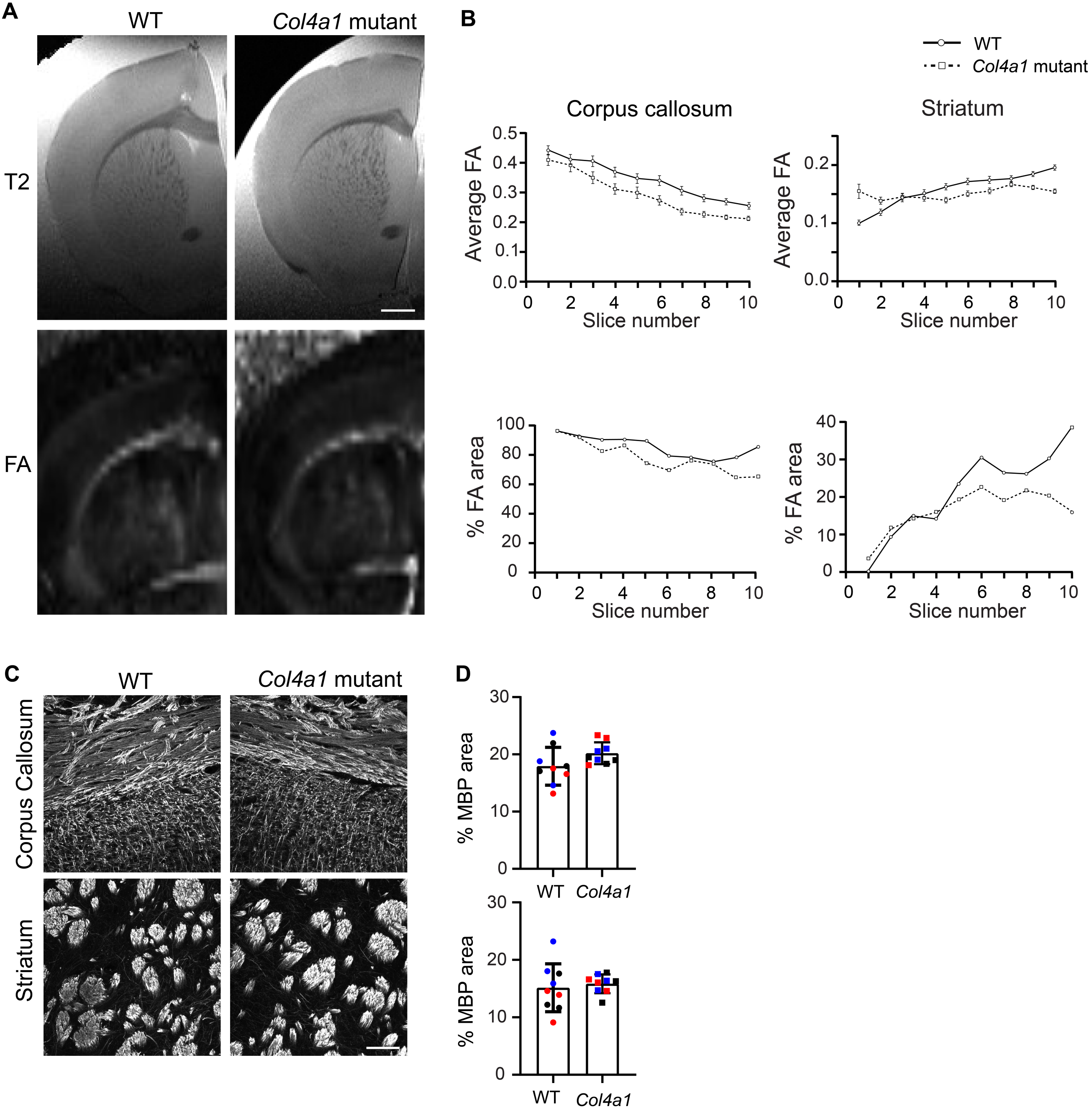

Supplementary Fig. 3

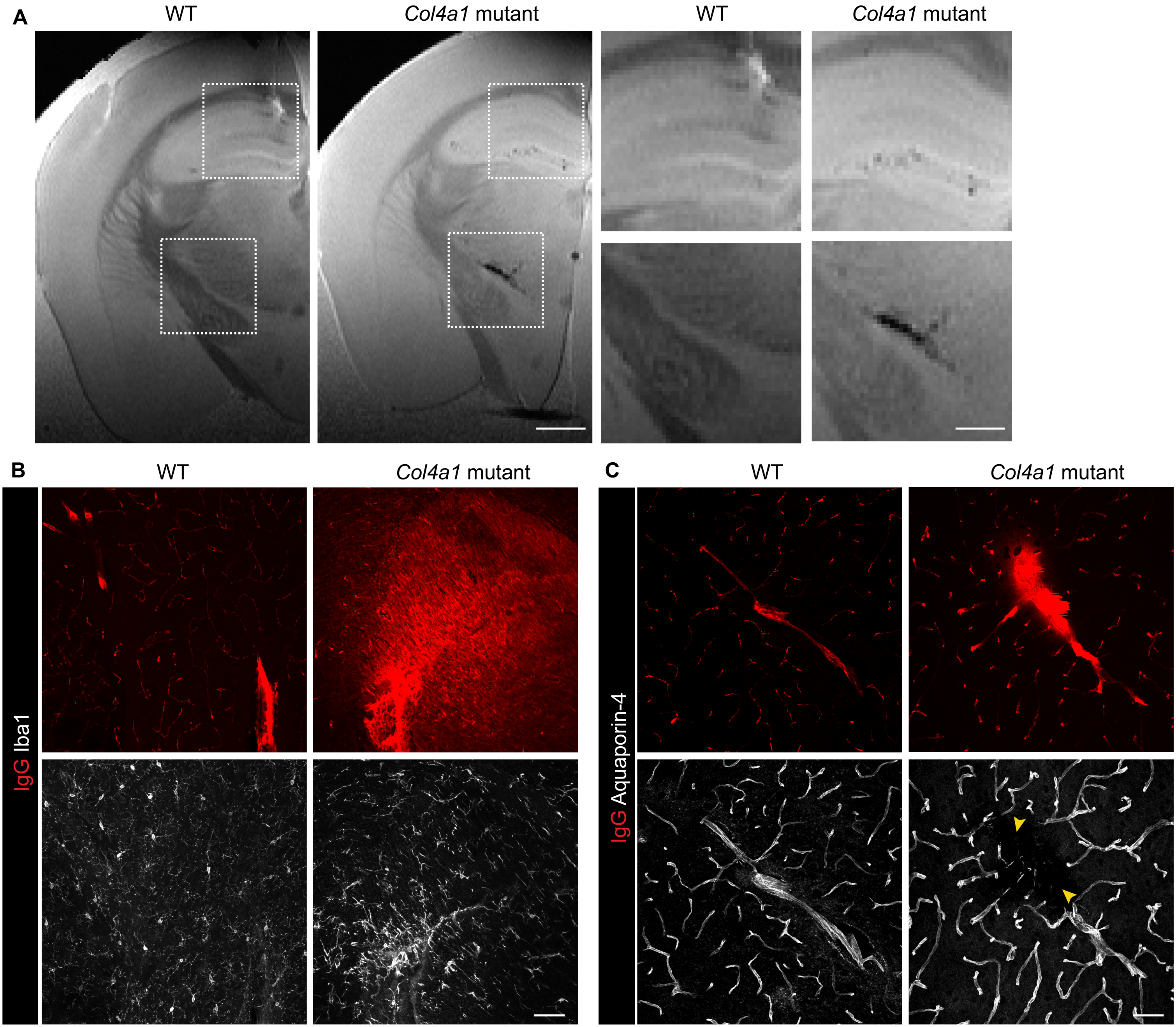

Supplementary Fig. 4

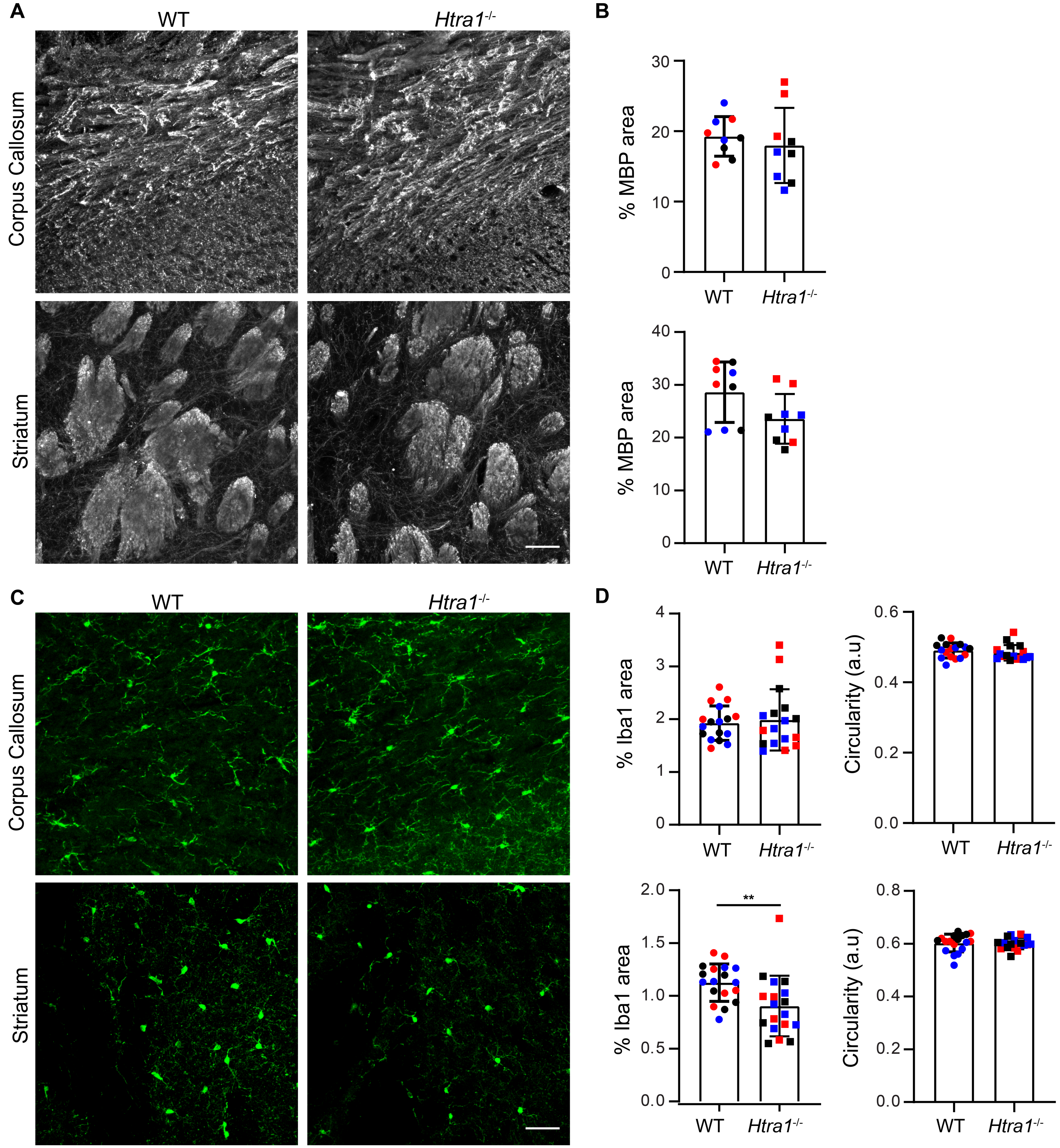

Supplementary Fig. 5

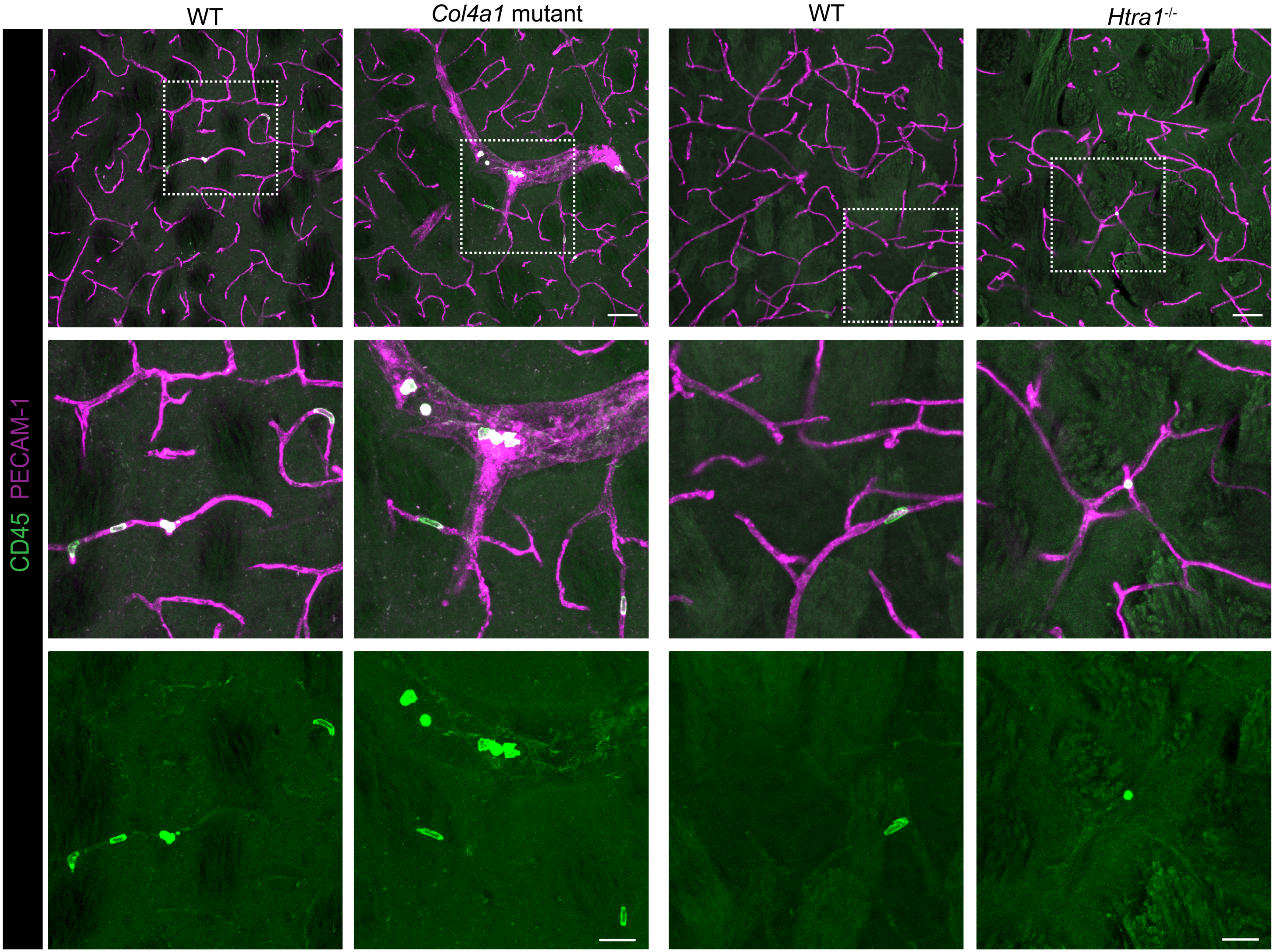

Supplementary Fig. 6

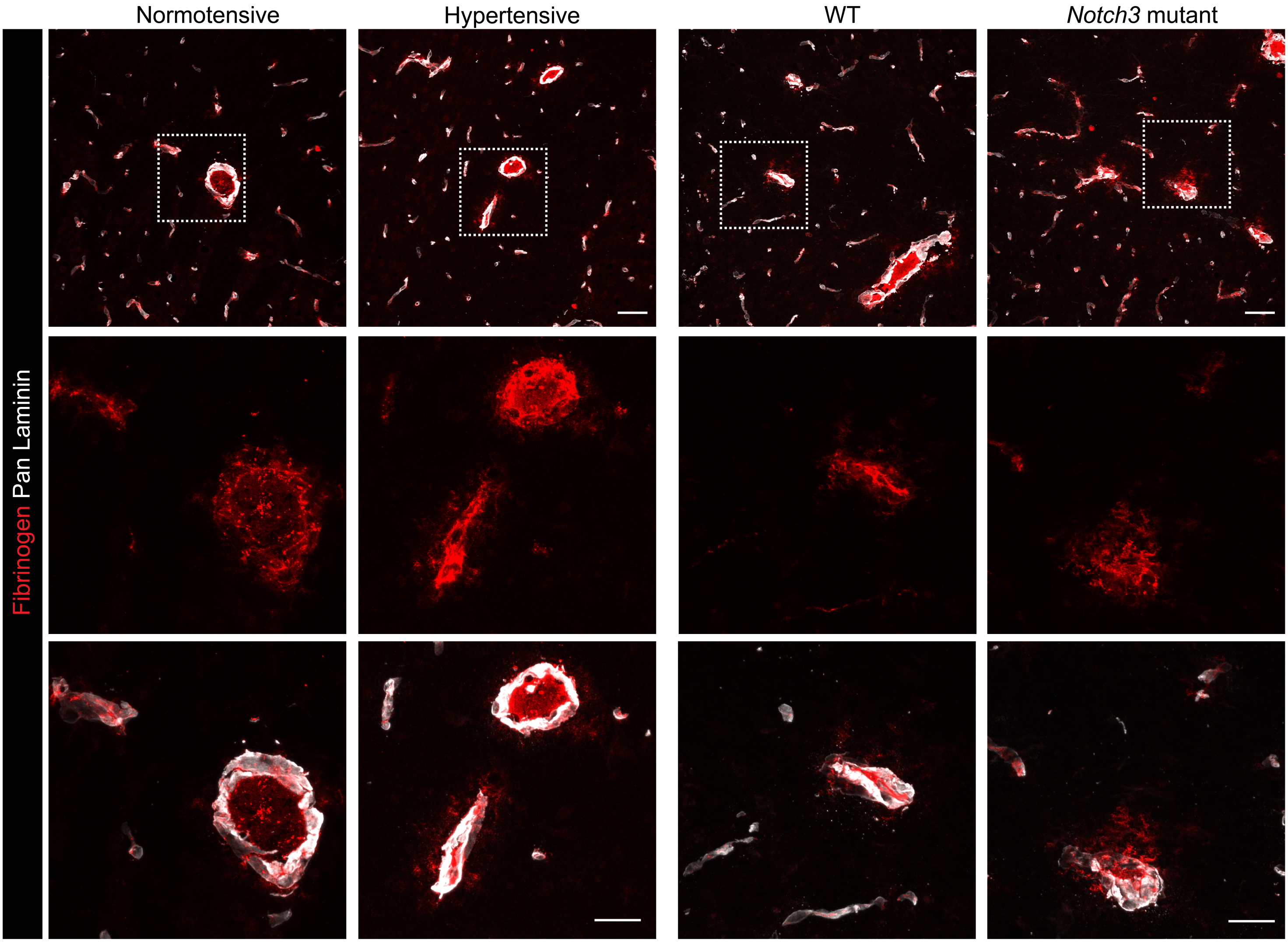

Supplementary Fig. 7

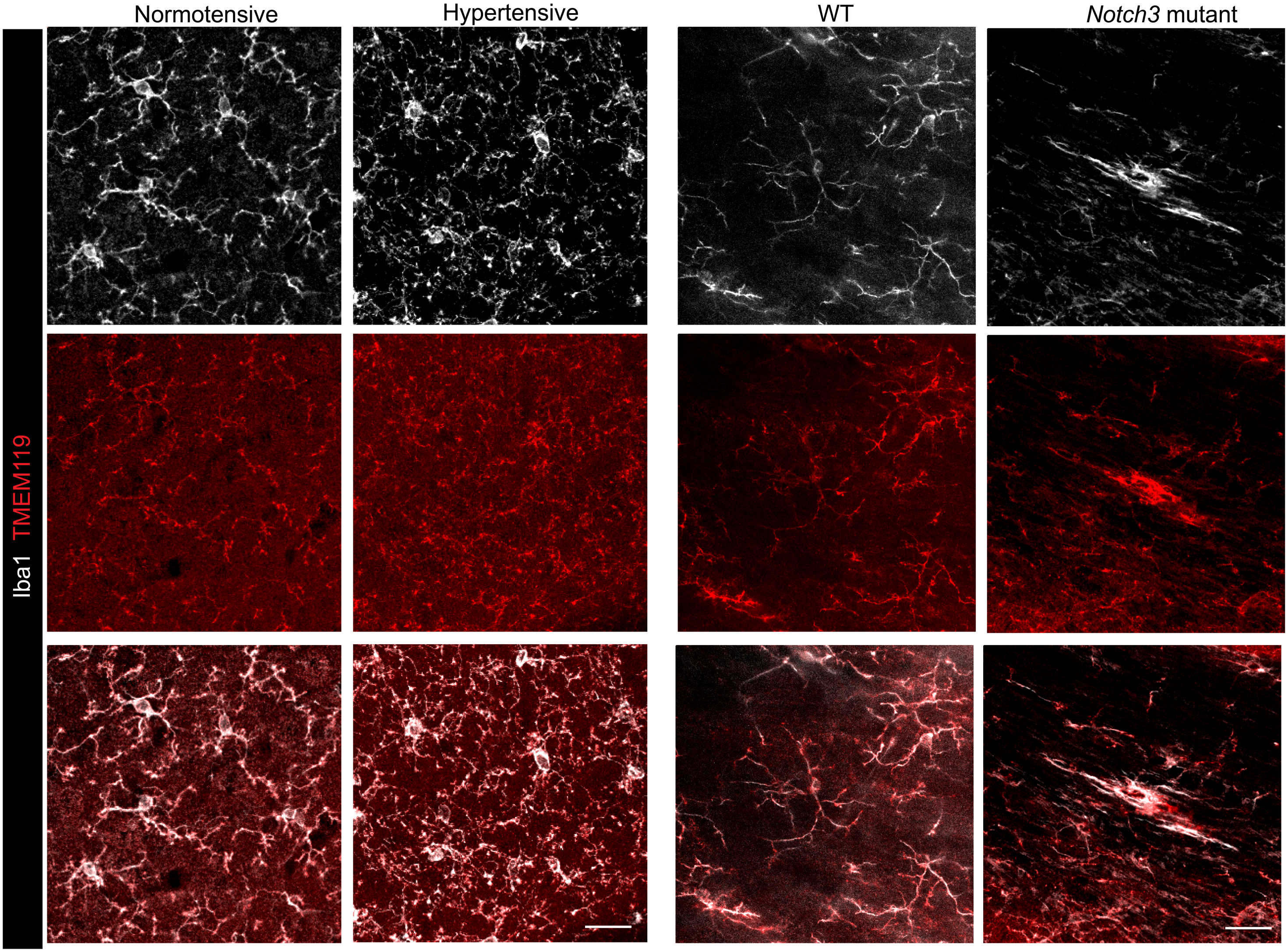

Supplementary Fig. 8

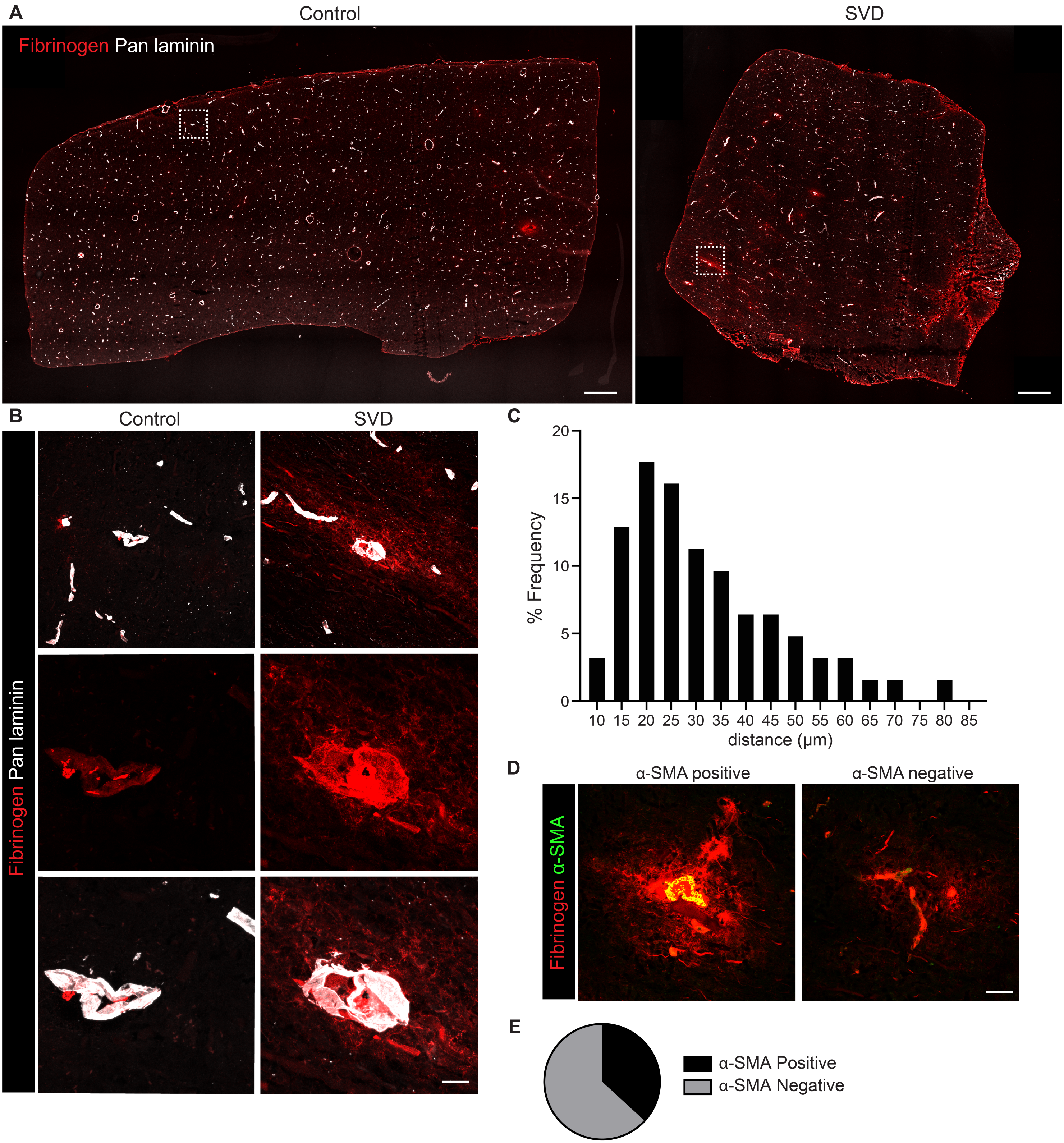

Supplementary Fig. 9

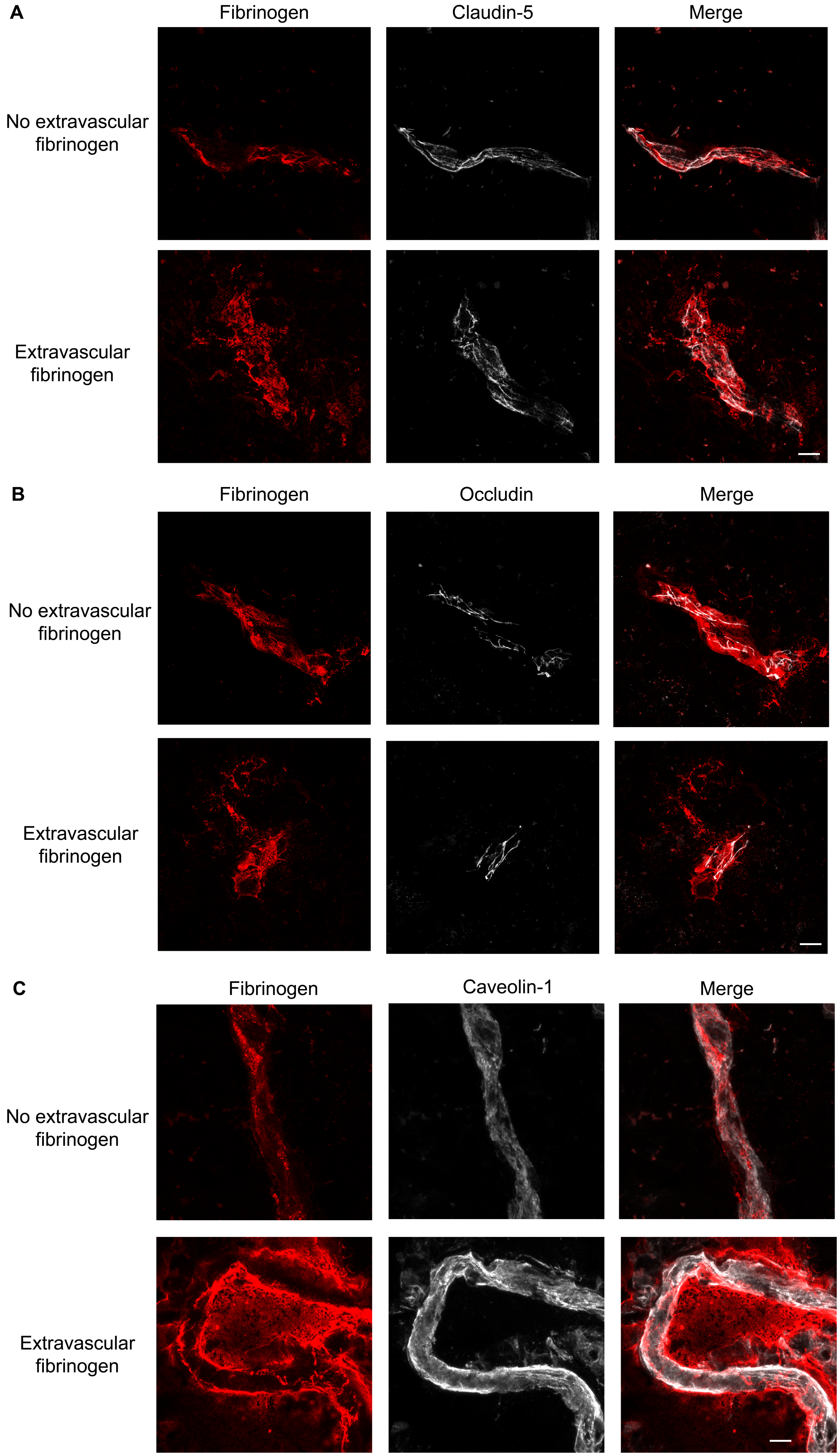

Supplement: Supplementary file 2 — Supplementary Figures [file NAN-50-e13015-s001.pdf]
